# Itaconate suppresses atherosclerosis by activating a Nrf2-dependent antiinflammatory response in macrophages in mice

**DOI:** 10.1172/JCI173034

**Published:** 2024-02-01

**Authors:** Jianrui Song, Yanling Zhang, Ryan A. Frieler, Anthony Andren, Sherri Wood, Daniel J. Tyrrell, Peter Sajjakulnukit, Jane C. Deng, Costas A. Lyssiotis, Richard M. Mortensen, Morgan Salmon, Daniel R. Goldstein

**Affiliations:** 1 Department of Internal Medicine, Division of Cardiovascular Medicine, University of Michigan, Ann Arbor, Michigan, USA.; 2 Department of Biochemistry and Molecular Biology, Soochow University Medical College, Suzhou, Jiangsu, China.; 3 Department of Molecular and Integrative Physiology, University of Michigan, Ann Arbor, Michigan, USA.; 4 Department of Pathology, Heersink School of Medicine, University of Alabama at Birmingham, Alabama, USA.; 5 University of Michigan Rogel Cancer Center,; 6 Graduate Program in Immunology, and; 7 Department of Internal Medicine, Division of Pulmonary and Critical Care Medicine, University of Michigan, Ann Arbor, Michigan, USA.; 8 Veterans Affairs Ann Arbor Healthcare System, Ann Arbor, Michigan, USA.; 9 Department of Internal Medicine, Division of Gastroenterology, University of Michigan Medical School, Ann Arbor, Michigan, USA.; 10 Department of Pharmacology,; 11 Department of Internal Medicine, Division of Metabolism, Endocrinology, and Diabetes,; 12 Department of Cardiac Surgery,; 13 Department of Microbiology and Immunology, University of Michigan, Ann Arbor, Michigan, USA.

**Keywords:** Cardiology, Inflammation, Atherosclerosis, Cardiovascular disease, Macrophages

## Abstract

Itaconate has emerged as a critical immunoregulatory metabolite. Here, we examined the therapeutic potential of itaconate in atherosclerosis. We found that both itaconate and the enzyme that synthesizes it, aconitate decarboxylase 1 (Acod1, also known as immune-responsive gene 1 [IRG1]), are upregulated during atherogenesis in mice. Deletion of *Acod1* in myeloid cells exacerbated inflammation and atherosclerosis in vivo and resulted in an elevated frequency of a specific subset of M1-polarized proinflammatory macrophages in the atherosclerotic aorta. Importantly, Acod1 levels were inversely correlated with clinical occlusion in atherosclerotic human aorta specimens. Treating mice with the itaconate derivative 4-octyl itaconate attenuated inflammation and atherosclerosis induced by high cholesterol. Mechanistically, we found that the antioxidant transcription factor, nuclear factor erythroid 2–related factor 2 (Nrf2), was required for itaconate to suppress macrophage activation induced by oxidized lipids in vitro and to decrease atherosclerotic lesion areas in vivo. Overall, our work shows that itaconate suppresses atherogenesis by inducing Nrf2-dependent inhibition of proinflammatory responses in macrophages. Activation of the itaconate pathway may represent an important approach to treat atherosclerosis.

## Abstract 2

<p>Itaconate suppresses atherogenesis by inhibition of the proinflammatory response via Nrf2 in mouse macrophages.&nbsp;</p>

## Graphical abstract

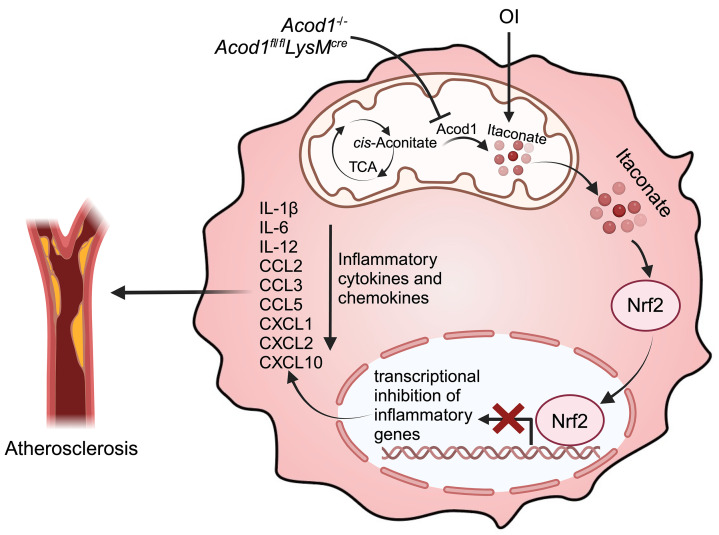

## Introduction

Itaconate has emerged as a compelling immunomodulatory metabolite produced in the tricarboxylic acid (TCA) cycle (1, 2). The enzyme *cis*-aconitate decarboxylase 1 (Acod1, also named Irg1) is responsible for itaconate synthesis and is highly expressed in macrophages. Studies of preclinical mouse models suggest that Acod1 and itaconate attenuate various noninfectious inflammatory conditions that involve macrophages, such as psoriasis (3), ischemia-reperfusion injury of both the brain and heart (4, 5), lung fibrosis (6), and abdominal aortic aneurysms (7). More recently, it was reported that Acod1 contributes to the immunosuppressive function of tumor-associated macrophages and diminishes the efficacy of cancer immunotherapy (8). Additionally, Acod1 was shown to suppress cardiac inflammation and fibrosis after acute myocardial infarction and chemotherapeutic drug exposure (9).

Itaconate is typically produced by macrophages to inhibit proinflammatory responses at several levels. These include inhibition of glycolysis, inhibition of succinate dehydrogenase and thus ROS generation by mitochondria, and suppression of the NLRP3 inflammasome (10, 11). Itaconate also upregulates antioxidant pathways, for instance, by positively regulating the nuclear factor erythroid 2–related factor 2 (Nrf2) transcription factor (12, 13) and activating transcription factor 3 (ATF3) (14). Ultimately, itaconate suppresses macrophages’ ability to produce several proinflammatory cytokines, whereas *Acod1*-deficient macrophages exhibit an increased production of such cytokines (4). Hence, previous studies suggest that itaconate acts as a brake to restrain macrophage inflammatory responses.

Macrophages are key drivers of atherosclerosis, one of the most common vascular metabolic diseases (15, 16). Whether itaconate affects the progression of atherosclerosis is not known. Here, we show that Acod1 and itaconate attenuate atherogenesis by inducing an antiinflammatory response via Nrf2. Overall, our work suggests the therapeutic potential of employing itaconate to treat atherosclerosis.

## Results

### Acod1 and itaconate accumulate during atherosclerosis.

To determine whether itaconate biosynthesis changes during atherogenesis, we examined the expression of Acod1 in mice with or without atherosclerosis. Briefly, we induced hypercholesterolemia and atherosclerosis by intraperitoneally injecting mice with an adeno-associated virus that overexpresses proprotein convertase subtilisin/kexin type 9 (PCSK9-AAV) and feeding them a Western diet (WD) for 10 weeks (17, 18). Nonatherosclerotic controls were administered vehicle and fed a standard laboratory chow diet.

**Figure 1:**
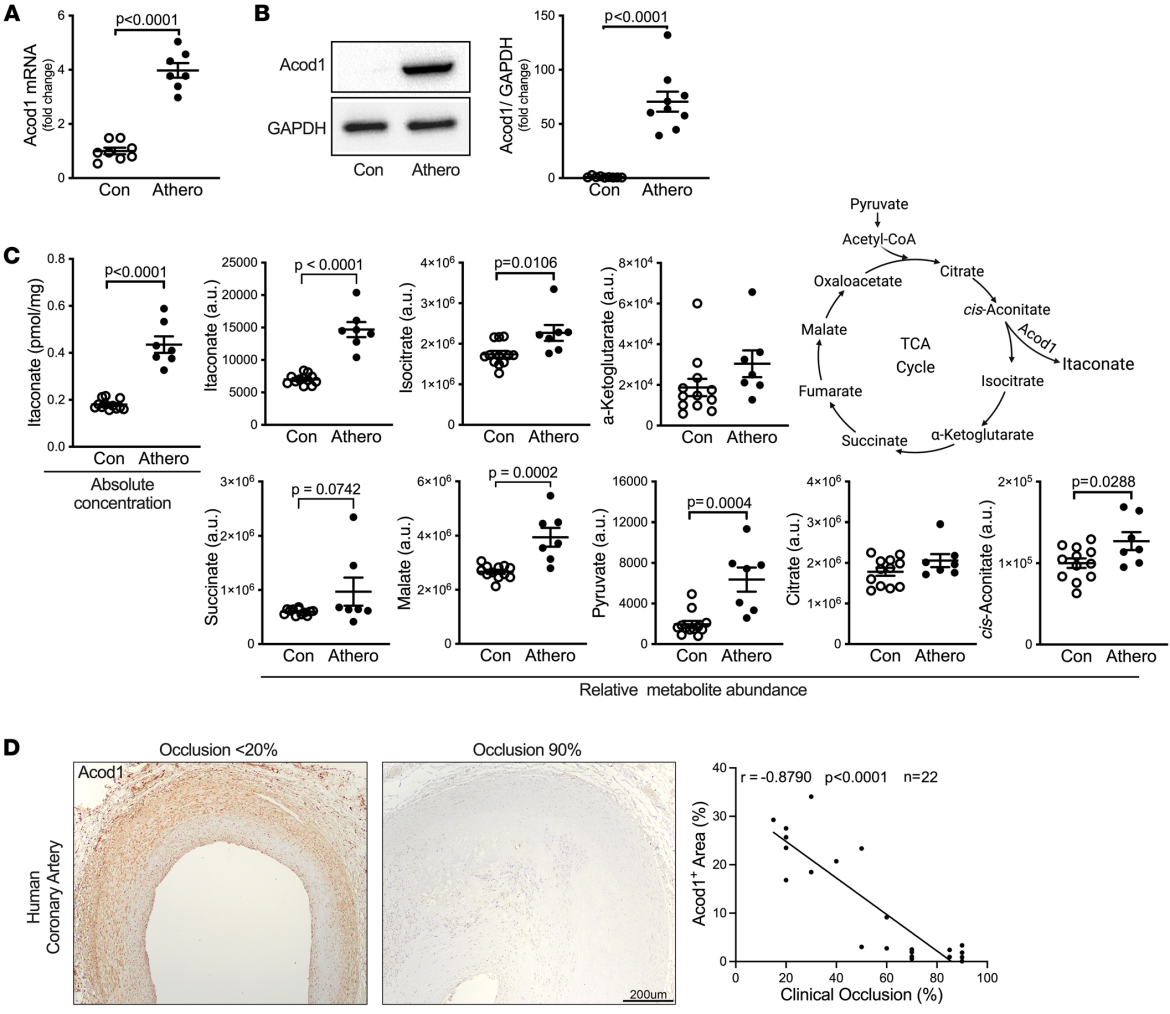
Acod1 expression and Itaconate production increase in atherosclerotic aorta. Atherosclerosis was induced by intraperitoneally injecting mice with PCSK9-AAV and feeding a Western diet (WD) for 10 weeks. (**A**) *Acod1* mRNA levels in control (Con, *n* = 8) and atherosclerotic (Athero, *n* = 7) aortas were measured by qRT-PCR. (**B**) Aorta lysates from Con and Athero mice were separated by gel electrophoresis and proteins were detected by Western blotting with the indicated antibodies. The quantification of Acod1 (*n* = 9/group) after normalization to GAPDH is shown on the right. (**C**) Relative abundance of TCA cycle metabolites (itaconate, isocitrate, α-ketoglutarate, succinate, malate, pyruvate, citrate, and *cis*-aconitate) was measured by metabolomics in nonatherosclerotic control (*n* = 12) and atherosclerotic (*n* = 7) aortas. a.u., arbitrary units based on MS peak area. The absolute concentrations of itaconate in aortas were also measured. (**D**) Representative images of anti-Acod1–stained human atherosclerotic coronary artery. Correlation between the percentage Acod1-positive area and clinical occlusion using 2-sided Pearson’s correlation analysis is shown on the right (*n* = 22). In **A**–**C**, results are presented as mean ± SEM, and unpaired, 2-tailed Student’s *t* test was used for statistical analysis.

We observed significantly increased levels of Acod1 mRNA and protein in the atherosclerotic aortas versus healthy control aortas (Figure 1, A and B). Consistent with the elevated abundance of Acod1, atherosclerotic aortas accumulated more itaconate than controls (*P* < 0.0001, Figure 1C). We examined other TCA cycle metabolites and found that isocitrate, malate, pyruvate, and *cis*-aconitate were slightly but significantly increased in atherosclerotic aorta, whereas α-ketoglutarate, succinate, and citrate were mildly but not significantly affected (Figure 1C).

To investigate Acod1 levels during atherogenesis in human patients, we collected human atherosclerotic coronary arteries from a deidentified repository and performed immunohistochemistry (IHC). Intriguingly, a correlation analysis between the clinical occlusion percentage and the Acod1-positive area showed that the occlusion was significantly negatively correlated with Acod1 staining (*P* < 0.0001, Figure 1D). These data suggest that Acod1 expression during atherogenesis mitigates occlusion.

Collectively, these results suggest that hypercholesterolemia and atherosclerosis lead to increased levels of Acod1 and itaconate, which in turn may help to attenuate atherogenesis.

### Acod1 deficiency exacerbates atherosclerosis in vivo.

To determine whether Acod1 plays a role in atherogenesis, we examined *Acod1^–/–^* C57BL/6N mice before and during hypercholesterolemia (19, 20). We verified that Acod1 protein was not detected in *Acod1^–/–^* aortas (Supplemental Figure 1A; supplemental material available online with this article; https://doi.org/10.1172/JCI173034DS1), and itaconate levels did not increase with atherogenesis in *Acod1^–/–^* aortas (Supplemental Figure 1B).

WT and *Acod1^–/–^* mice displayed comparable plasma cholesterol levels throughout the hypercholesterolemia regimen (Supplemental Figure 2A). To investigate glucose metabolism, we performed glucose tolerance tests (GTT) and insulin tolerance tests (ITT). *Acod1^–/–^* mice initially had lower glucose tolerance and insulin sensitivity than WT mice; however, hypercholesterolemia reduced the glucose tolerance and insulin sensitivity of WT mice over time, such that both genotypes displayed similarly compromised glucose metabolism from 3 weeks of hypercholesterolemia onwards (Supplemental Figure 2C). WT and *Acod1^–/–^* mice had similar body weights that increased due to hypercholesterolemia (Supplemental Figure 2D). Fat mass also increased with hypercholesterolemia, and although *Acod1^–/–^* mice initially had a higher fat mass than WT mice, this difference was eliminated after week 6 of hypercholesterolemia (Supplemental Figure 2D). WT and *Acod1^–/–^* mice had similar liver weights, gonadal white adipose tissue (GWAT) weights, liver lipid droplet accumulation, and GWAT adipocyte sizes after 10 weeks of hypercholesterolemia (Supplemental Figure 2B). Importantly, hypercholesterolemia resulted in elevated levels of itaconate in the aortas of WT mice but not *Acod1^–/–^* mice (*P* < 0.0001) (Supplemental Figure 2E).

**Figure 2:**
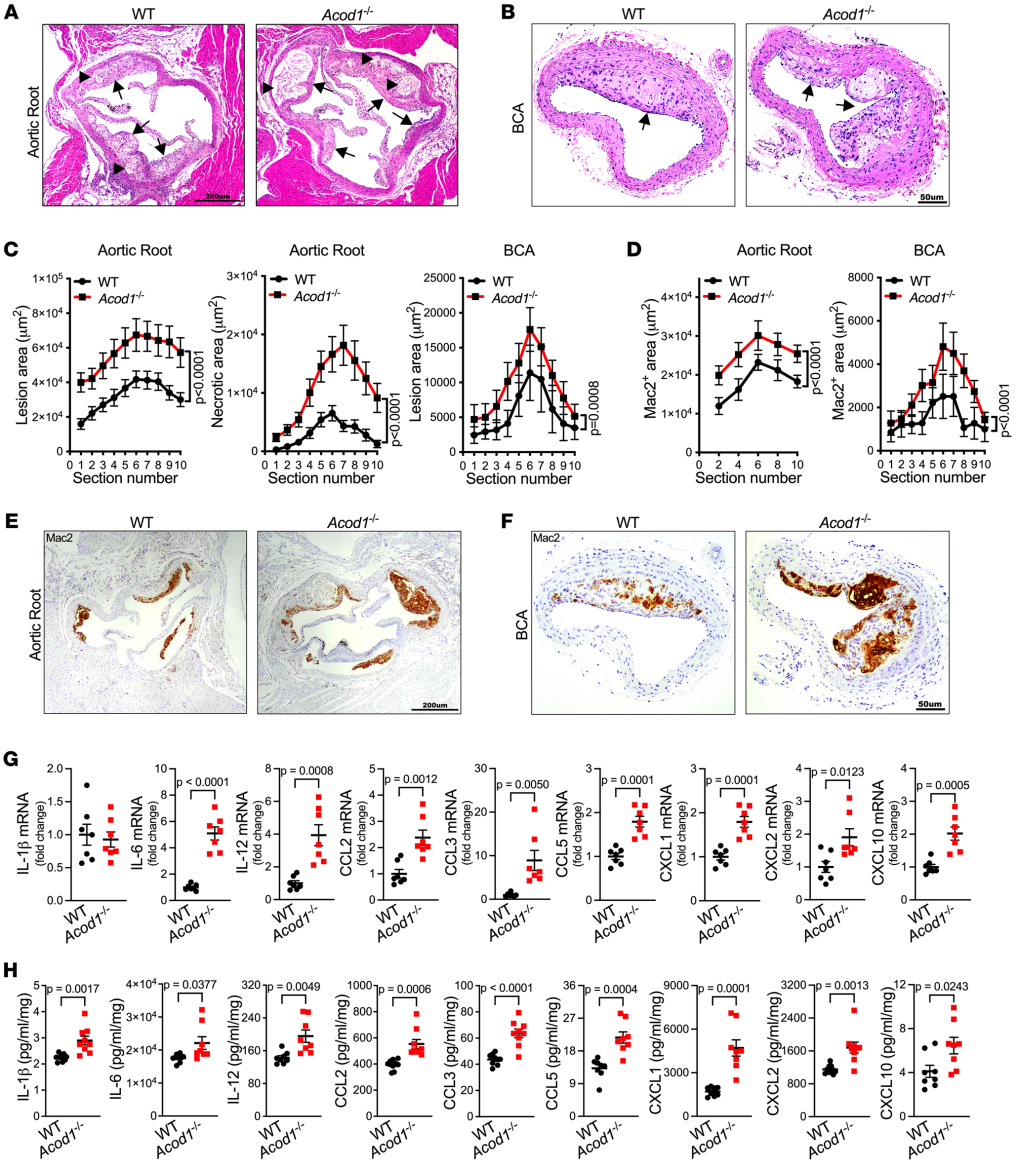
Acod1 deficiency promotes atherosclerosis by enhancing inflammation. WT and *Acod1^–/–^* mice were induced to become atherosclerotic via PCSK9-AAV administration followed by 10-week Western diet. (**A** and **B**) Representative images of H&E-stained (**A**) aortic root and (**B**) brachiocephalic artery (BCA) sections of WT and *Acod1^–/–^* mice. Arrows indicate atherosclerotic lesions and arrowheads indicate necrotic cores. (**C**) The quantifications of lesion area and necrotic area in each section of aortic root (*n* = 23–24/group) and BCA (*n* = 10/group) are shown. (**D**–**F**) The quantification of Mac2-positive area in each section of aortic root (*n* = 13/group) and BCA (*n* = 10/group) of atherosclerotic WT and *Acod1^–/–^* mice is shown (**D**), with representative images of anti-Mac2–stained (**E**) aortic root and (**F**) BCA sections. (**G** and **H**) The inflammatory cytokines and chemokines’ (**G**) gene expression in atherosclerotic aorta and (**H**) protein levels in tissue culture medium of atherosclerotic aortas from WT and *Acod1^–/–^* mice, including IL-1β, IL-6, IL-12, CCL2, CCL3, CCL5, CXCL1, CXCL2, and CXCL10, were measured by qRT-PCR (*n* = 7/group) and multiplex assay (*n* = 8–9/group), respectively. Results are presented as mean ± SEM. Two-way ANOVA followed by Tukey’s post hoc test was used in **C** and **D** and unpaired, 2-tailed Student’s *t* test was used in **G** and **H** for statistical analysis. Scale bars: 200 μm (**A** and **E**) and 50 μm (**B** and **F**).

We hypothesized that Acod1 and itaconate help to attenuate atherogenesis. Indeed, we observed larger atherosclerotic lesions in both the aortic root and brachiocephalic artery (BCA) of *Acod1^–/–^* mice compared with WT after 10 weeks of hypercholesterolemia (Figure 2, A–C). Additionally, compared with atherosclerotic WT mice, atherosclerotic *Acod1^–/–^* mice exhibited a more than 3-fold increase in necrotic area in the aortic root (Figure 2, A and C), a surrogate of plaque instability in the murine model of atherosclerosis (21).

Atherosclerotic progression involves dysfunctional turnover of the extracellular matrix, partly due to an imbalance of matrix metalloproteinases (MMPs) and tissue inhibitors of metalloproteinases (TIMPs) (22). We examined the expression of select MMPs and TIMPs, and found that MMP9 and MMP12 were upregulated in atherosclerotic aortas from *Acod1^–/–^* mice versus WT, at both mRNA and protein levels (Supplemental Figure 3, A and B). These MMPs have been found to promote plaque instability and increase atherosclerotic burden (22, 23), consistent with more severe atherogenesis in *Acod1^–/–^* versus WT mice.

### Acod1 deficiency increases inflammation during atherogenesis.

To determine whether Acod1 deficiency affects inflammation in response to atherogenesis, we measured macrophage infiltration in the aorta, which is another surrogate marker of plaque instability (24). By IHC, we observed increased staining for the macrophage marker Mac2 in the aortic root and BCA of atherosclerotic *Acod1^–/–^* mice compared with WT (Figure 2, D–F). In a complementary analysis, we enumerated macrophages and neutrophils via flow cytometry. Consistent with the observations above, the proportion and absolute number of macrophages were elevated in the atherosclerotic aorta of *Acod1^–/–^* compared with WT mice, whereas the proportion of neutrophils in the aorta was not affected (Supplemental Figure 4A). We infer that Acod1 deficiency increases macrophage infiltration in the aorta during atherogenesis.

Additionally, we measured circulating monocytes, which are a critical supply for atherosclerotic plaque macrophages (15, 25, 26), and circulating neutrophils, which are involved in monocyte recruitment (27). Intriguingly, we observed an increase in both peripheral neutrophils and peripheral monocytes (including the Ly6C^hi^ inflammatory and Ly6C^lo^ patrolling subpopulations) in atherosclerotic *Acod1^–/–^* mice compared with WT (Supplemental Figure 4B). Notably, levels of peripheral neutrophils and monocytes were initially similar in *Acod1^–/–^* and WT mice, but both were increased in *Acod1^–/–^* mice compared with WT after 8 to 10 weeks of hypercholesterolemia, coincident with atherosclerosis (Supplemental Figure 4C). Thus, the absence of Acod1 during the development of atherosclerosis is associated with elevated levels of circulating neutrophils and monocytes.

To further investigate the impact of Acod1 deficiency on inflammation during atherogenesis, we measured the aortic gene expression of the following 9 proinflammatory cytokines and chemokines that have been implicated in atherosclerosis: IL-1β, IL-6, IL-12, CCL2, CCL3, CCL5, CXCL1, CXCL2, and CXCL10 (25, 28, 29). All but IL-1β mRNA showed increased mRNA levels in atherosclerotic aorta from *Acod1^–/–^* compared with WT mice (Figure 2G). Furthermore, all 9 corresponding proteins were secreted at higher levels in culture supernatant from atherosclerotic aortas of *Acod1^–/–^* compared with WT mice (Figure 2H). We also examined the antiinflammatory cytokines IL-4, IL-10, and TGF-β (30–32). Both IL-4 and IL-10 were slightly but significantly increased in culture supernatant of atherosclerotic aorta from *Acod1^–/–^* mice compared with WT, whereas TGF-β isoforms were not significantly different between genotypes (Supplemental Figure 4D).

Finally, we analyzed the expression of genes involved in the resolution of inflammation mediated by lipids. We found that the expression of 12/15-lipoxygenase (12/15-LO), which plays a protective role in atherogenesis (33), was decreased by approximately 50% in atherosclerotic aortas from *Acod1^–/–^* mice versus WT (Supplemental Figure 4E). In contrast, COX-2, which promotes atherogenesis (34), exhibited an approximately 3-fold increase in atherosclerotic aortas from *Acod1^–/–^* mice versus WT (Supplemental Figure 4E).

Taken together, our results indicate that constitutive genetic inactivation of *Acod1* exacerbates hypercholesterolemia-induced inflammation and atherogenesis, associated with increased lesion size, elevated macrophage infiltration, elevated peripheral neutrophils and monocytes, and higher expression of proinflammatory cytokines and chemokines.

### Acod1 deficiency promotes the proinflammatory polarization of macrophages during atherogenesis.

To determine how loss of Acod1 affects specific immune cell types during atherogenesis, we obtained plaque-containing atherosclerotic aorta tissues from WT and *Acod1^–/–^* mice and performed single-cell RNA sequencing (scRNA-seq). We detected many different types of immune cells and stromal cells (Supplemental Figure 5A), as expected.

**Figure 3:**
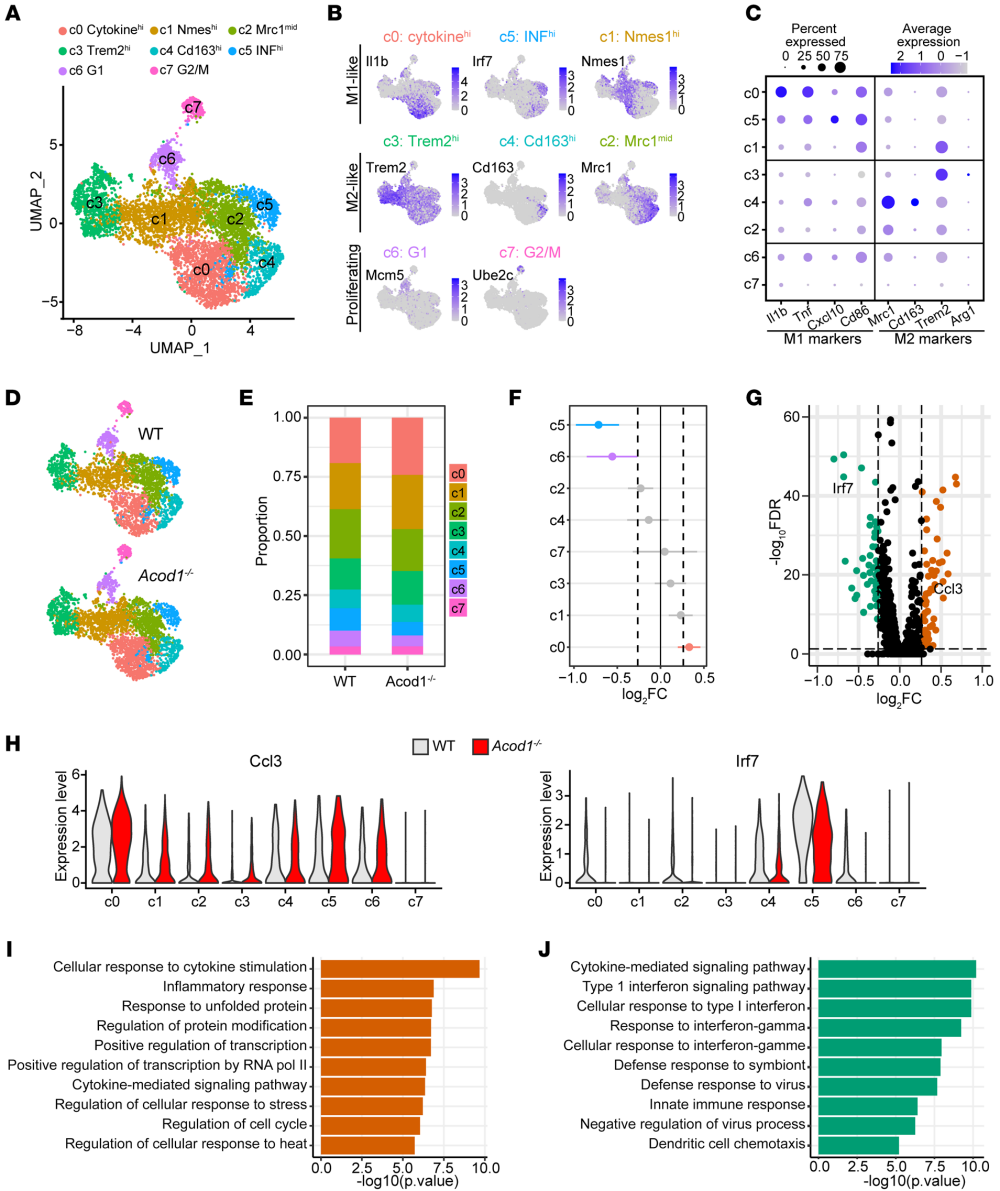
Single-cell analysis of macrophages within the atherosclerotic aorta of WT and *Acod1^–/–^* mice. WT and *Acod1^–/–^* mice were induced to become atherosclerotic via PCSK9-AAV administration followed by 10-week Western diet. (**A**) UMAP plot showing 8 different macrophage subpopulations revealed by scRNA-seq. (**B**) The expression of representative signature genes from each macrophage subpopulation was overlaid on the UMAP plot. Color intensity indicates normalized expression levels as shown for each gene. (**C**) The expression of M1-like and M2-like marker genes in each macrophage subpopulation was determined. The size of the dots indicates the percentage of cells expressing the gene of interest, while the intensity of the color indicates expression levels. (**D**) UMAP plots of macrophages from atherosclerotic WT and *Acod1^–/–^* aortas. Clusters are colored as in **A**. (**E**) The proportion of macrophage subpopulations from atherosclerotic WT and *Acod1^–/–^* aortas. (**F**) Differential abundance testing of changes in the proportion of macrophage subpopulations in atherosclerotic *Acod1^–/–^* aortas. Clusters that passed the threshold of adjusted *P* values < 0.05 and log_2_FC > 1.2 were deemed significant and colored. FC, fold change. (**G**) Volcano plot showing differentially expressed genes in macrophages from atherosclerotic *Acod1^–/–^* aortas. Up- and downregulated genes are colored orange and green, respectively. FDR, false discovery rate. (**H**) Violin plots showing the expression of 2 representative genes, *Ccl3* and *Irf7*, that were differentially expressed between WT and *Acod1^–/–^* across all macrophage subpopulations. (**I** and **J**) Gene ontology analysis of (**I**) up- and (**J**) downregulated genes in macrophages from atherosclerotic *Acod1^–/–^* aortas.

Macrophages are a main constituent of atherosclerotic plaques and known to highly express *Acod1* (4, 14, 35); thus, our initial analysis focused on this cell type. After quality control, we profiled 3050 WT and 3617 *Acod1^–/–^* macrophages, which is comparable to previous single-cell studies on atherosclerotic macrophages (36, 37). Consistent with prior reports (36, 37), plaque macrophages exhibited strong heterogeneity that clustered into 8 subsets (clusters 0–7, abbreviated as c0–c7) (Figure 3A). Clusters c0, c5, and c1 were M1-like macrophages and generally expressed high levels of M1 markers such as *Il1b*, *Tnf*, *Cxcl10*, and/or *Cd86* (Figure 3, B and C). Clusters c3, c4, and c2 were M2-like macrophages and had high expression of typical M2 markers such as *Trem2*, *Mrc1*, *Cd163*, and/or *Arg1* (Figure 3, B and C). These 6 clusters were spread continuously on the UMAP plot (Figure 3A), suggesting a spectrum of macrophage activation status in vivo. The other 2 clusters, c6 and c7, were separated from the main populations and represented proliferating macrophages in G_1_ and G_2_/M phases, with low expression of M1 and M2 markers (Figure 3, A–C). All 8 clusters were present in both WT and *Acod1^–/–^* atherosclerotic aortas (Figure 3D).

Cluster c0 macrophages displayed unusually high cytokine and chemokine expression (i.e., *Il1b*, *Il1a*, *Tnf*, *Cxcl2*, *Cxcl1*, *Cxcl10*, *Ccl2*, *Ccl3*, *Ccl4*, *Ccl12*, and *Cxcr4*) compared with other clusters (Supplemental Table 1). This macrophage subset was previously described as “chemokine^hi^ macrophages” (36) and “inflammatory macrophages” (37). Importantly, we detected a higher proportion of c0 M1-like macrophages in atherosclerotic plaques from *Acod1^–/–^* compared with WT mice (Figure 3, E and F, and Supplemental Table 2). In contrast, the proportion of c5 M1-like macrophages was decreased in *Acod1^–/–^* compared with WT plaques (Figure 3, E and F, and Supplemental Table 2). The c5 macrophages had a uniquely high type I IFN response (i.e., *Ifit3*, *Ifit2*, *Ifit3b*, *Ifit1*, *Irf7*, *Ifi206*, *Ifi213*, *Ifi44*, *Ifi211*, *Ifi205*, *Ifi47*, *Ifi209*, *Isg15*, and *Isg20*), which corresponded to previously described “IFN signature^hi^ macrophages” (36). Type I IFN macrophages have been shown to exert both pro- and antiinflammatory roles during atherosclerosis (38). The relative proportion of M2-like macrophages appeared roughly unchanged between WT and *Acod1^–/–^* (Figure 3, E and F, and Supplemental Table 2). Macrophage proliferation within plaques can contribute to atherosclerosis, especially at later stages (39–41), but c7 (G_2_/M macrophages) was comparable between WT and *Acod1^–/–^*, whereas c6 (G_1_ macrophages) was decreased in *Acod1^–/–^* atherosclerotic aortas (Figure 3, E and F, and Supplemental Table 2).

We determined the differentially expressed genes between macrophages from WT and *Acod1^–/–^* atherosclerotic aortas at the single-cell level (Figure 3, G–J, and Supplemental Table 3). Consistent with the results above, genes encoding proinflammatory cytokines and chemokines, and inflammatory response–related genes, were upregulated in macrophages from *Acod1^–/–^* versus WT plaques, whereas type I IFN response genes were downregulated (Figure 3, G–J, and Supplemental Table 3). Collectively, these results suggest that Acod1 deficiency leads to an increased frequency of c0 M1-like macrophages with augmented proinflammatory polarization in atherosclerotic plaques.

We observed 2 subsets of monocytes in the atherosclerotic plaques, corresponding to classical Ly6c^hi^ and nonclassical Ly6c^lo^ monocytes. Compared with WT, the aortic plaques from *Acod1^–/–^* mice had a higher proportion of Ly6c^lo^ monocytes (Supplemental Figure 5, A–D), which could reflect an increased conversion of Ly6c^hi^ monocytes into inflammatory macrophages.

Consistent with other scRNA-seq studies (42, 43), we detected several types of dendritic cells (DCs) (Supplemental Figure 5, E–G). In addition, we also detected T and B lymphocytes, NK cells, and type 2 innate lymphoid cells (ILC2s) in atherosclerotic aortas. Interestingly, the proportion of mature/migratory DCs was increased in atherosclerotic aortas from *Acod1^–/–^* mice compared with WT (Supplemental Figure 5, E–G). This DC subtype was shown to accumulate during advanced phases of atherosclerosis (44). Importantly, CD8^+^ T cells displayed an increased frequency among T lymphocytes from *Acod1^–/–^* aortic plaques compared with WT (Supplemental Figure 5, H–J)*,* consistent with their role in promoting atherosclerosis (44, 45). Notably, many stress-related chaperones were upregulated at the single-cell level in DCs, T cells, B cells, and ILC2s from *Acod1^–/–^* atherosclerotic plaques compared with WT, whereas type II immune response genes were downregulated in ILC2s and neutrophils from *Acod1^–/–^* plaques (Supplemental Figure 6, Supplemental Figure 7, and Supplemental Table 4). Multiple inflammatory cytokines (CCL4, CCL5, CCL11, CXCL2, and CXCL12) had elevated expression in fibroblasts from *Acod1^–/–^* plaques compared with WT (Supplemental Figure 8 and Supplemental Table 4). Overall, these results are consistent with a highly inflammatory microenvironment in *Acod1^–/–^* atherosclerotic aortas.

### Acod1 in macrophages protects mice from atherogenesis.

**Figure 4:**
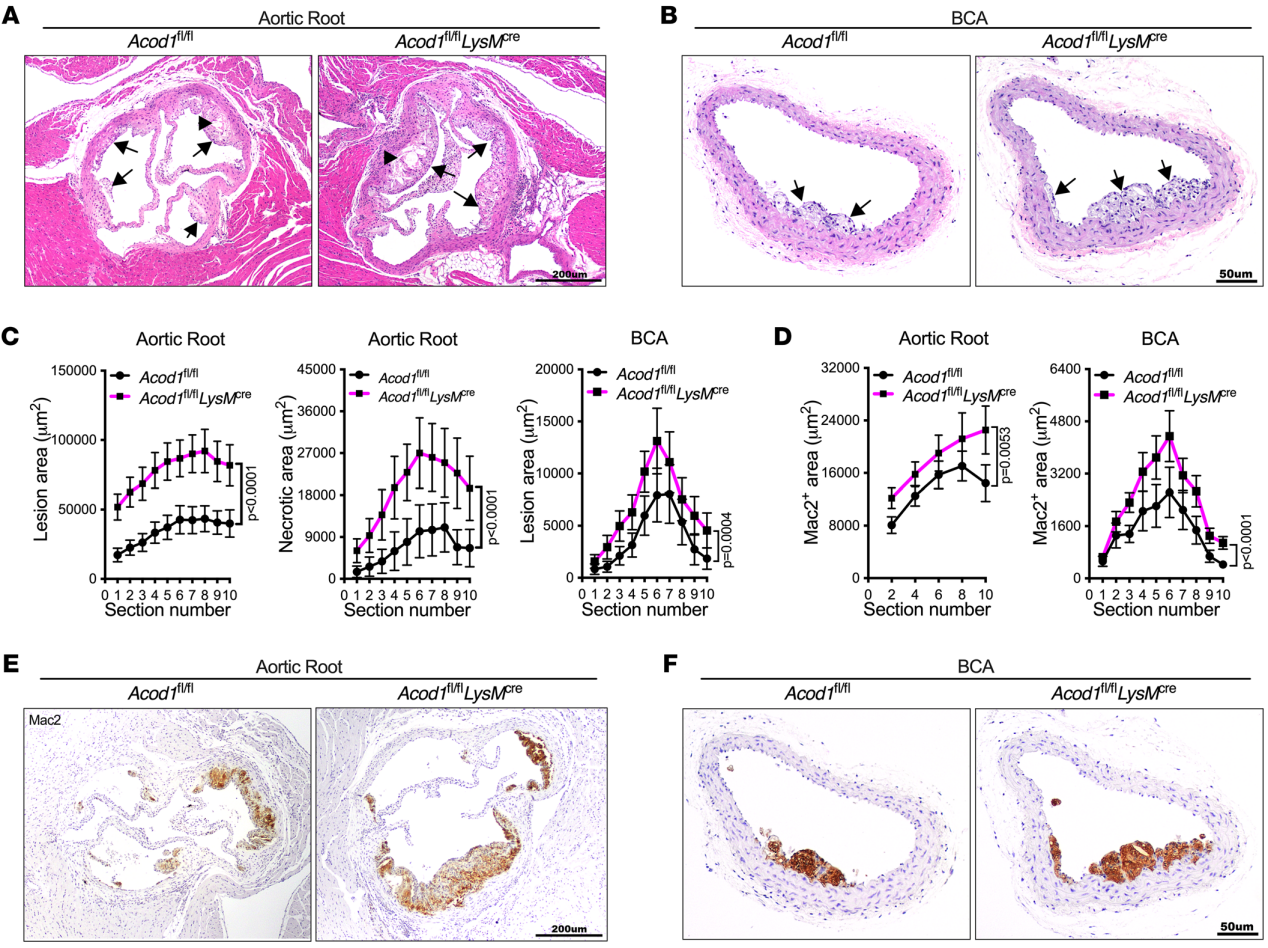
Acod1 deficiency in myeloid cells confers increased atherogenesis and macrophage infiltration. *Acod1^fl/fl^* and *Acod1^fl/fl^* *LysM*^cre^ mice were induced to become atherosclerotic via PCSK9-AAV administration followed by 10-week Western diet. (**A** and **B**) Representative images of H&E-stained (**A**) aortic root and (**B**) brachiocephalic artery (BCA) sections of atherosclerotic *Acod1^fl/fl^* and *Acod1^fl/fl^* *LysM*^cre^ mice. Arrows indicate atherosclerotic lesions and arrowheads indicate necrotic cores. (**C**) The quantifications of lesion area and necrotic area in each section of aortic root (*n* = 17–20/group) and BCA (*n* = 17–19/group) are shown. (**D**–**F**) The quantification of Mac2-positive area in each section of aortic root (*n* = 9/group) and BCA (*n* = 11–13/group) of atherosclerotic *Acod1^fl/fl^* and *Acod1^fl/fl^* *LysM*^cre^ mice is shown (**D**), along with representative images of anti-Mac2–stained (**E**) aortic root and (**F**) BCA sections. Results are shown as mean ± SEM. Two-way ANOVA followed by Tukey’s post hoc test was used for statistical analysis. *P* values indicate the main effect of the comparison. Scale bars: 200 μm (**A** and **E**) and 50 μm (**B** and **F**).

To investigate the role of Acod1 in macrophages during atherogenesis, we used myeloid-specific lysosomal-M (LysM)-Cre mice to generate conditional *Acod1*-knockout mice (*Acod1^fl/fl^* *LysM^cre^*). We induced hypercholesterolemia and atherosclerosis in *Acod1^fl/fl^* *LysM^cre^* and in *Acod1^fl/fl^* littermate controls. We did not detect a difference in fasting cholesterol between atherosclerotic *Acod1^fl/fl^* *LysM^cre^* and *Acod1^fl/fl^* mice (Supplemental Figure 9A). However, we observed an approximately 2-fold increase in the atherosclerotic lesion sizes in both the aortic root and BCA of *Acod1^fl/fl^* *LysM^cre^* mice compared with control littermates, and an almost 3-fold increase in aortic root necrotic core size (Figure 4, A–C). These increases are comparable to those caused by ubiquitous Acod1 deficiency (Figure 2, A–C). Mac2 staining was also higher in both the aortic root and BCA of atherosclerotic *Acod1^fl/fl^* *LysM^cre^* mice compared with the *Acod1^fl/fl^* controls (Figure 4, D–F). Relative to control *Acod1^fl/fl^* aortas, *Acod1^fl/fl^* *LysM^cre^* aortas displayed increased infiltration of macrophages and increased expression of the macrophage markers F4/80, CD68, and CD64, whereas neutrophil infiltration and the levels of neutrophil markers like Mpo, Elane, and S100a8 were unchanged (Supplemental Figure 9, B and C). Taken together, our results show that Acod1 in macrophages protects mice from atherogenesis during hypercholesterolemia.

### The itaconate derivative 4-octyl itaconate attenuates atherosclerosis, mitochondrial dysfunction, and inflammation in WT mice.

**Figure 5:**
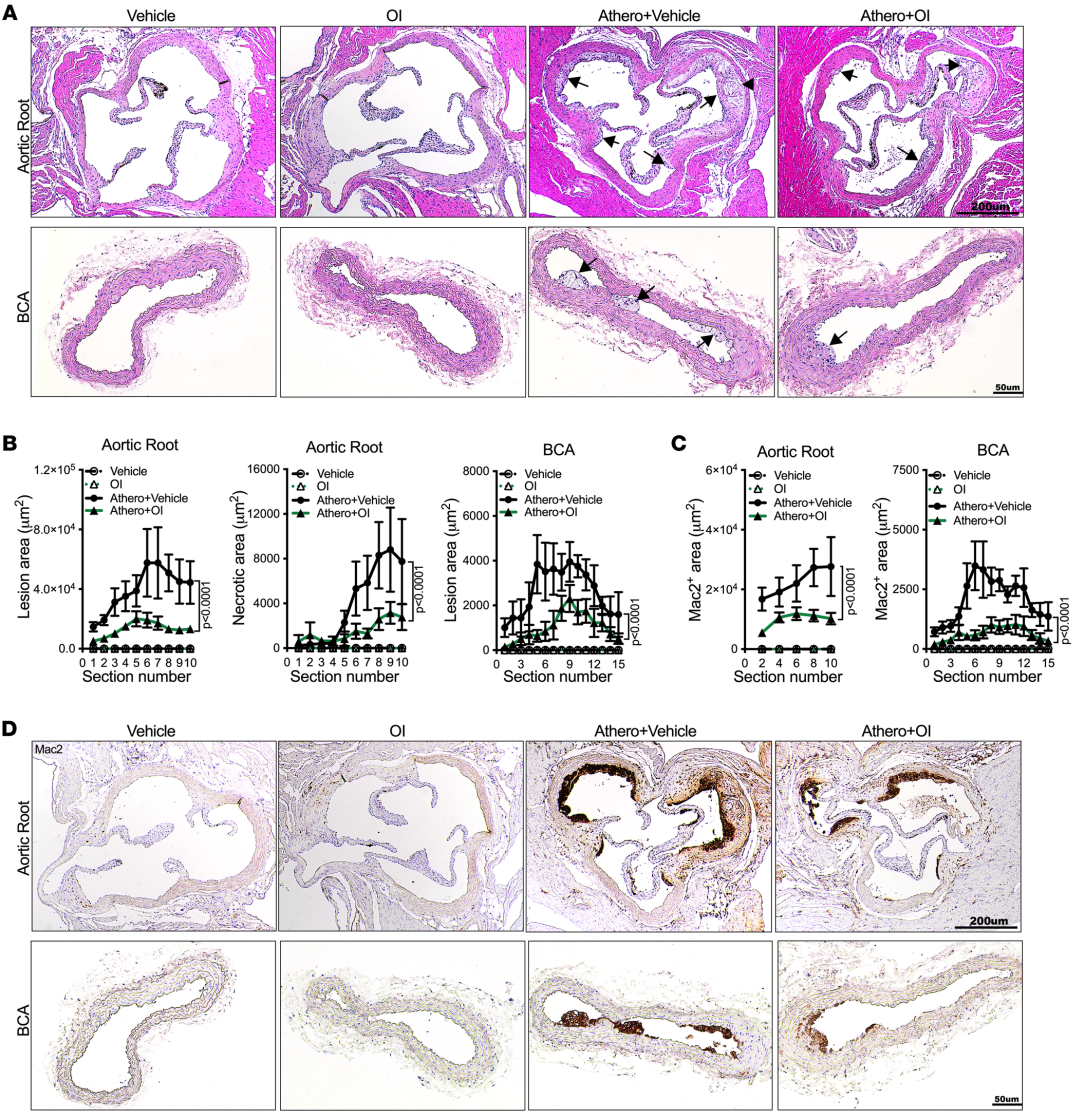
Itaconate derivative 4-octyl itaconate inhibits atherogenesis. (**A**) Representative images of H&E-stained aortic root and brachiocephalic artery (BCA) sections of mice with indicated treatment. Vehicle, vehicle control; OI, 4-octyl itaconate; Athero+Vehicle, atherosclerosis and vehicle; Athero+OI, atherosclerosis and 4-octyl itaconate. Arrows indicate atherosclerotic lesions and arrowheads indicate necrotic cores. (**B** and **C**) The quantifications of lesion area and necrotic area (**B**) as well as Mac2-positive area (**C**) in each section of aortic root and BCA from indicated mice are shown (*n* = 7–8/group). (**D**) Representative images of anti-Mac2–stained aortic root and BCA sections of mice with indicated treatment. Results are shown as mean ± SEM. Two-way ANOVA followed by Tukey’s post hoc test was used for statistical analysis. *P* values indicate the main effect of the comparison between Athero+Vehicle vs. Athero+OI. Scale bars: 200 μm (top row in **A** and **D**) and 50 μm (bottom row in **A** and **D**).

4-Octyl itaconate (OI) is an esterified derivative of itaconate that has been employed to mimic the in vivo biological effects of itaconate (14). To test the potential therapeutic efficacy of itaconate in atherosclerosis, we administered OI or vehicle via intraperitoneal injection twice per week for 10 weeks in WT mice with or without hypercholesterolemia. We found that the atherosclerotic lesion area, necrotic area, and Mac2-positive area within the aortic root and BCA were all reduced in atherosclerotic WT mice that received OI versus vehicle (Figure 5). Thus, OI treatment attenuated atherogenesis caused by hypercholesterolemia in WT mice.

To confirm the effectiveness of OI, we analyzed mitochondrial function by performing a Seahorse assay on peritoneal and aortic macrophages isolated from these 4 groups of mice. Compared with macrophages from healthy mice, those from atherosclerotic mice displayed an increased extracellular acidification rate (ECAR) and decreased oxygen consumption rate (OCR) (Supplemental Figure 10A), likely due to upregulated glycolysis (46). Notably, this compromise in mitochondrial function was significantly attenuated by OI treatment (Supplemental Figure 10A). We infer that OI treatment significantly inhibited the metabolic reprogramming and the shift from oxidative phosphorylation to glycolysis in macrophages during atherosclerosis. Moreover, we found that OI treatment attenuated the increased ROS production in macrophages during atherogenesis (Supplemental Figure 10B). These results are consistent with changes in cellular metabolism upon exposure to OI (4, 12, 47).

**Figure 6:**
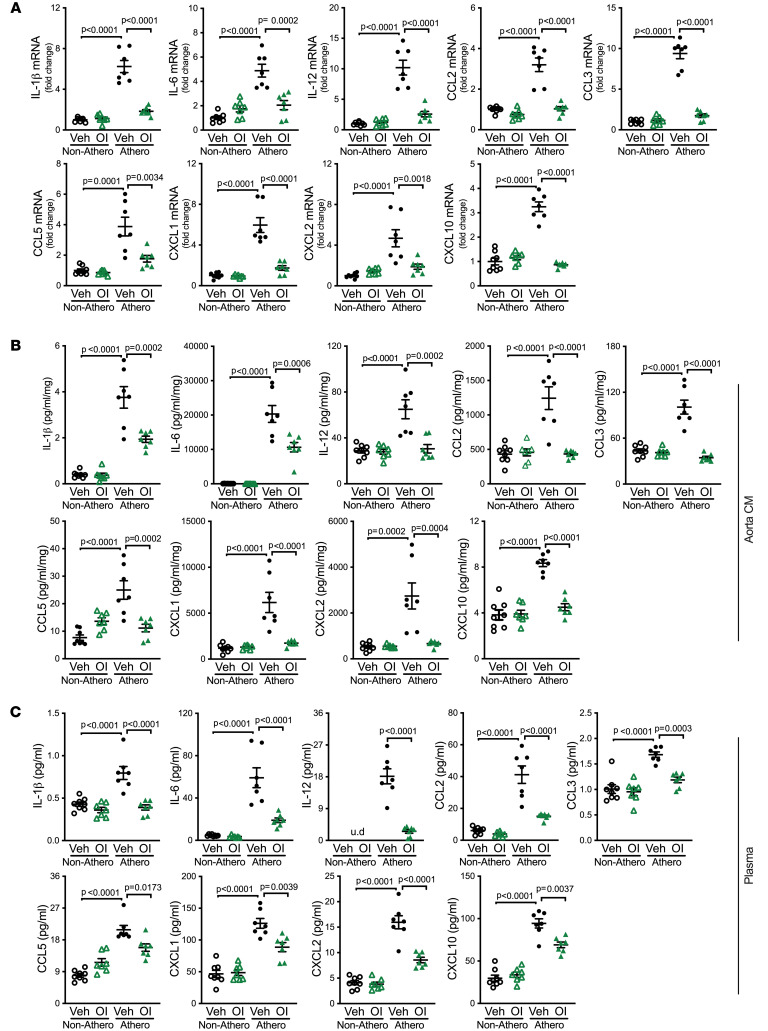
4-Octyl itaconate attenuates inflammation caused by atherosclerosis. WT mice were subjected to the following treatments: 4-octyl itaconate (OI) only, atherosclerosis (Athero) only, and OI plus Athero. Vehicle was used as the control for OI, and mice without atherosclerosis (Non-Athero) were used as control mice. (**A**) Gene expression of inflammatory cytokines and chemokines, including IL-1β, IL-6, IL-12, CCL2, CCL3, CCL5, CXCL1, CXCL2, and CXCL10, in aortas of indicated mice were measured by qRT-PCR (*n* = 7–8/group). (**B** and **C**) The protein levels of inflammatory cytokines and chemokines, including IL-1β, IL-6, IL-12, CCL2, CCL3, CCL5, CXCL1, CXCL2, and CXCL10 in (**B**) tissue culture medium (CM) of aortas or (**C**) plasma of indicated mice were determined by multiplex assay (*n* = 7–8/group). u.d., undetectable. Protein levels in aorta CM were normalized to tissue weight for analysis. Results are presented as mean ± SEM. Two-way ANOVA followed by Tukey’s post hoc test was used for statistical analysis.

Moreover, OI treatment restored the levels of all 9 proinflammatory cytokines and chemokines at the mRNA level in atherosclerotic aortas of WT mice (Figure 6A), as well as at the protein level in the media of cultured atherosclerotic aortas (Figure 6B) and in plasma (Figure 6C). The levels of the antiinflammatory cytokines IL-4, IL-10, and TGF-β1 in atherosclerotic aortas were also restored by OI treatment of WT mice during atherogenesis (Supplemental Figure 11A). OI treatment reduced the expression of COX-2, chemokine like receptor 1 (Cmklr1), and formyl peptide receptor 2 (Fpr2), but increased the expression of 12/15-LO in atherogenic aortas (Supplemental Figure 11B), consistent with their respective pro- or antiinflammatory roles during the resolution of inflammation (33, 34, 48–50). OI treatment also suppressed the increased expression of MMP9, MMP12, and TIMP1 during atherogenesis (Supplemental Figure 11, C and D). Thus, we conclude that treatment with the itaconate derivative OI diminishes atherosclerosis and inflammation in WT mice.

### Nrf2 is required for the itaconate-mediated inhibition of atherogenesis in mice.

**Figure 7:**
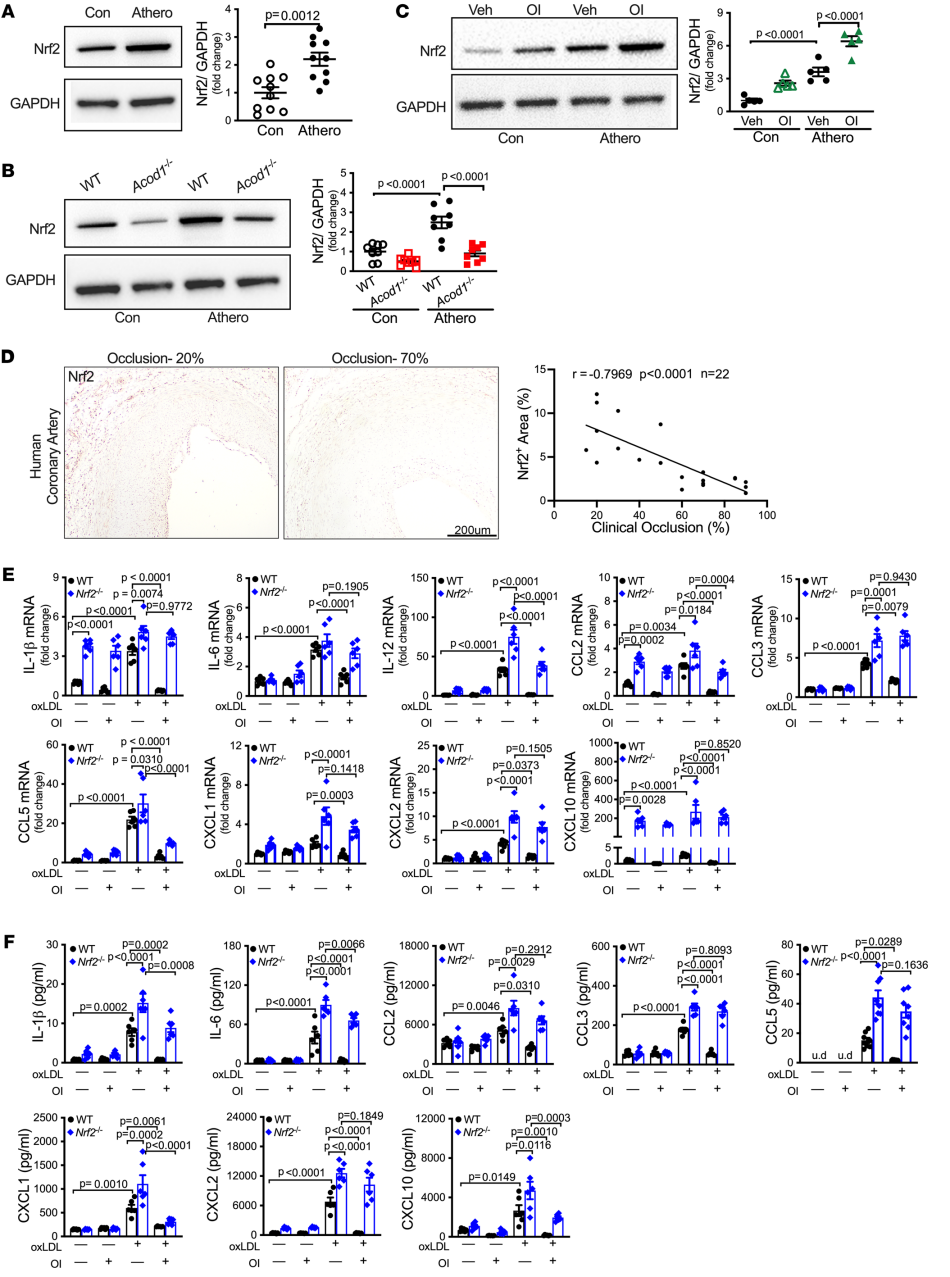
Nrf2 signaling is importantly involved in suppressing atherogenesis and inflammation mediated by itaconate. (**A**) Whole-cell lysates were extracted from aortas of mice with or without atherosclerosis, and the protein level of Nrf2 was determined by Western blotting. The quantification is shown on the right (*n* = 10/group). (**B**) Aorta lysates from WT and *Acod1^–/–^* mice with or without atherosclerosis were extracted, and Nrf2 protein level was determined by Western blotting. The quantifications of Nrf2 (*n* = 8/group) that were normalized to GAPDH are shown on the right. (**C**) Aorta lysates from WT mice (with or without atherosclerosis) that were subjected to 4-octyl itaconate (OI) administration or vehicle control (Veh) were immunoblotted against Nrf2. Quantification is shown on the right (*n* = 5/group). GAPDH was used as loading control in **A**–**C**. Con, control; Athero, atherosclerosis. (**D**) Representative images of anti-Nrf2–stained human atherosclerotic coronary artery. Scale bar: 200 μm. Correlation between the percentage Nrf2-positive area and clinical occlusion using 2-sided Pearson’s correlation analysis is shown on the right (*n* = 22). (**E** and **F**) Bone marrow–derived macrophages (BMDMs) from WT and *Nrf2^–/–^* mice were treated with or without OI (250 μM), followed by exposure to oxLDL (100 μg/mL). Vehicle was used as control. Cells and culture medium supernatant were collected at the end of experiment, and RNA was extracted from the cells. The inflammatory cytokines’ and chemokines’ (**E**) gene expression in those BMDMs and (**F**) protein levels in the culture media, including IL-1β, IL-6, IL-12, CCL2, CCL3, CCL5, CXCL1, CXCL2, and CXCL10, were measured by qRT-PCR and multiplex assay, respectively (*n* = 6/group). u.d., undetectable. Results are presented as mean ± SEM. Unpaired, 2-tailed Student’s *t* test was used in **A** and 2-way ANOVA followed by Tukey’s post hoc test was used in **B**, **C**, **E**, and **F** for statistical analysis.

Our results show that Acod1 in macrophages protects mice from atherogenesis. Given that itaconate activates Nrf2 signaling to protect against inflammation and oxidative stress in bone marrow–derived macrophages (BMDMs) and tissues (12, 51, 52), we hypothesized that Acod1 and itaconate inhibit atherogenesis and inflammation, at least in part, by activating Nrf2 signaling. To test this, first we measured Nrf2 protein levels in WT nonatherosclerotic and atherosclerotic aortas by Western blotting. Nrf2 protein levels were almost 3-fold higher in atherosclerotic versus nonatherosclerotic aortas in WT mice (Figure 7A), indicating that Nrf2 levels increase during atherogenesis. Compared with WT aortas, *Acod1^–/–^* aortas displayed reduced levels of Nrf2 protein, particularly after atherogenesis (Figure 7B). Intriguingly, WT mice treated with OI displayed elevated levels of Nrf2 protein in nonatherosclerotic and especially in atherosclerotic aortas (Figure 7C). Moreover, we observed a negative correlation between Nrf2 abundance and the extent of occlusion in human coronary arteries by IHC (Figure 7D), suggesting that Nrf2 inhibits atherogenesis.

To determine whether Nrf2 helps to suppress atherogenesis mediated by Acod1 and itaconate, we examined *Nrf2^–/–^* mice. Atherogenesis was partially attenuated in *Nrf2^–/–^* mice compared with WT mice (Supplemental Figure 12), which is consistent with the phenotype observed in *ApoE^–/–^* *Nrf2^–/–^* mice (53), probably due to the role of Nrf2 in CD36 expression and foam cell formation (53–55). Importantly, however, although OI treatment significantly (*P* < 0.0001) decreased the lesion area and necrotic area in the aortic root and BCA in WT mice, it had no impact on these phenotypes in *Nrf2^–/–^* mice (Supplemental Figure 12). We infer that Nrf2 is required for the itaconate-mediated suppression of atherogenesis in WT mice.

### Nrf2 contributes to the itaconate-mediated suppression of macrophage proinflammatory responses.

Nrf2 suppresses macrophage inflammatory responses (56). To investigate the role of Nrf2 in macrophages during atherogenesis, we calculated the activity of the Nrf2 signaling pathway in the scRNA-seq data set from WT and *Acod1^–/–^* atherosclerotic plaques. We used the UCell algorithm (57), which scores the relative gene expression of Nrf2 target genes in each cell. We found that Nrf2 activity was slightly but significantly lower in macrophages from *Acod1^–/–^* atherosclerotic plaques compared with WT counterparts (Supplemental Figure 13A). As expected, Nrf2 activity was higher in antiinflammatory M2-like Trem2^hi^ macrophages compared with the other macrophage subpopulations (Supplemental Figure 13B).

We further examined Nrf2 responses in BMDMs exposed to oxidized low-density lipoprotein cholesterol (oxLDL), a major activator of atherogenesis (58–60), and/or OI. We measured the expression of the Nrf2 target genes *Hmox1*, *Nqo1*, and *Prdx1* and found that oxLDL induced the expression of *Hmox1* and *Nqo1* in WT BMDMs, with exposure to OI or OI plus oxLDL resulting in even stronger upregulation of all 3 Nrf2 target genes (Supplemental Figure 14). Nrf2 target genes were not upregulated in *Nrf2^–/–^* BMDMs under any condition (Supplemental Figure 14), as expected.

We next measured the gene expression of the 9 proinflammatory cytokines and chemokines, and found that all except CXCL1 were significantly upregulated in WT BMDMs exposed to oxLDL, and OI treatment restored their expression (Figure 7E). Many of these genes were also induced in *Nrf2^–/–^* BMDMs exposed to oxLDL, but the rescue by OI treatment was attenuated (IL-12, CCL2, and CCL5) or eliminated (e.g., IL-1β, IL-6, CCL3, CXCL1, CXCL2, and CXCL10; Figure 7E). Similarly, the levels of secreted proinflammatory cytokines and chemokines were elevated in WT BMDMs exposed to oxLDL, and this proinflammatory effect was dampened by cotreatment with OI (Figure 7F). Again, exposing *Nrf2^–/–^* BMDMs to oxLDL also elevated the level of secreted proinflammatory cytokines and chemokines, but the rescue by OI treatment was attenuated or eliminated (Figure 7F). Similar to OI, itaconate itself was also able to suppress oxLDL-mediated induction of the proinflammatory cytokines and chemokines (Supplemental Figure 15). We infer that itaconate activates Nrf2 in macrophages, which in turn mitigates a proinflammatory response.

## Discussion

Atherosclerosis is a chronic vascular disease that develops due to a failure to resolve inflammation within the arterial wall (61, 62). Typically, molecules that enhance the resolution of inflammation are lipid mediators (61–63), more specifically products derived from arachidonic acid, docosahexaenoic acid, or eicosapentaenoic acid (61).

In our study, we found that the immunoregulatory byproduct of the TCA cycle, itaconate, increases within the arterial wall during atherogenesis. Loss of the enzyme that synthesizes itaconate, Acod1, promoted atherogenesis, whereas delivery of the itaconate derivative OI, which enhances the effect of itaconate, suppressed atherosclerosis in WT mice. Collectively, these results reveal that itaconate suppresses atherogenesis caused by hypercholesterolemia. Our work suggests that itaconate production in the myeloid lineage, including macrophages, contributes to suppressing atherogenesis. During atherosclerosis, itaconate activates Nrf2, which downregulates the expression and secretion of proinflammatory cytokines and chemokines, thus restraining macrophage infiltration and proinflammatory polarization.

Acod1 and itaconate were both increased in atherosclerotic aortas. Likewise, Acod1 and itaconate were also increased in ocular bacterial infection (51) and idiopathic pulmonary fibrosis (64) in mice. However, itaconate levels were lower in bronchoalveolar lavage from pulmonary fibrosis patients compared with healthy volunteers (64). We found that Acod1 levels negatively correlated with clinical occlusion of the coronary artery in humans. Based on these data, we propose that inflammation initially induces an antiinflammatory response with subsequent itaconate production to dampen the inflammatory response. Pathogenesis arises both in experimental models and clinically when this antiinflammatory response is compromised, leading to worsening inflammation. We found that administering the itaconate derivative OI diminished atherosclerotic lesions in mice, consistent with the protective role of itaconate in other inflammatory diseases (10, 12, 47, 51, 52). In the future, it would be interesting to examine whether exogenous itaconate administration reduces atherosclerosis, given the difference between itaconate and its derivatives (65).

We found that multiple proinflammatory cytokines and chemokines were downregulated by OI treatment but upregulated by Acod1 deficiency during atherosclerosis, which is consistent with their effects on atherogenesis and with other studies (10, 20, 47, 51, 66). OI treatment decreased the levels of inflammatory cytokines and chemokines not only in the aorta, but also systemically in plasma. Given that we administered OI systemically via intraperitoneal injection, it may target cells throughout the body and result in a systemic decrease in inflammation. Additionally, OI-mediated inhibition of inflammation and atherogenesis would in turn decrease the release of inflammatory cytokines and chemokines into the circulation. The protective roles of itaconate and its derivatives against inflammation are supported by other studies. For instance, the proinflammatory factors IL-1β, IL-6, IL-12, CCL2, CCL3, and CXCL1 were also increased in the lungs of *Acod1^–/–^* compared with WT mice after *Mycobacterium* *tuberculosis* infection (20). Both IL-1β and IL-6 were increased by Acod1 deficiency, but decreased by OI treatment, in mouse retina during bacterial endophthalmitis (51). Serum (47) and peritoneal (10) IL-1β and IL-6 were also reduced by OI treatment of a murine model of lethal endotoxemia and peritonitis. CXCL10 levels in culture supernatant were decreased by itaconate treatment of lung tissue after influenza infection (66). Overall, itaconate and its derivatives appear to be a potent antiinflammatory therapeutic in multiple disease models.

Moreover, itaconate has been implicated in resolving inflammation. OI treatment of human BMDMs reduced COX-2 and MMP8 but increased TGF-β1, indicating that it promotes a wound-resolving phenotype (67). In our study, OI treatment of mice decreased COX-2, MMP8, MMP9, and MMP12 but increased 12/15-LO and TGF-β1, supporting a potential proresolving role in atherogenesis. Conversely, Acod1 deficiency resulted in decreased 12/15-LO, but increased COX-2, MMP8, MMP9, and MMP12. TGF-β1 did not show a significant change in *Acod1^–/–^* mice, which may reflect a stronger effect of exogenous itaconate or its derivative compared with endogenous itaconate. Itaconate was also shown to drive the resolution of pulmonary fibrosis (68) and allergen-induced airway inflammation (69), underscoring the therapeutic potential of targeting this pathway.

Our results indicate that myeloid-specific expression of Acod1 protects mice from atherosclerosis, as *Acod1^fl/fl^* *LysM^cre^* mice demonstrated aggravated atherogenesis compared with the *Acod1^fl/fl^* control. Our scRNA-seq data further reinforce the importance of macrophages in itaconate-mediated inflammatory blockade during atherogenesis. The c0 macrophage subset, named cytokine^hi^ M1-like macrophages, was significantly increased in *Acod1^–/–^* atherosclerotic aortas compared with WT. These macrophages express high levels of proinflammatory cytokines and chemokines and their receptors, including IL-1β, IL-1α, TNF-α, CCL2, CCL3, CCL4, CCL12, CXCL1, CXCL2, CXCL10, and CXCR4. Similarly, Acod1 in peritoneal tissue–resident macrophages was shown to be a potential therapeutic target for peritoneal tumors, as specifically silencing *Acod1* in these cells reduces peritoneal tumor burden (70). In addition, adoptive transfer of WT but not *Acod1^–/–^* monocyte–derived airway macrophages into the airway of *Acod1^–/–^* mice improved the outcome of bleomycin-induced pulmonary fibrosis (64), which further supports the importance of Acod1-expressing macrophages. As LysM is expressed in myeloid cells, including macrophages, developing a more specific Cre driver would help to precisely determine the exact identity of the itaconate-producing myeloid cells that suppress atherogenesis. More refined macrophage-specific Cre drivers are yet to be developed (71).

It should be noted that although macrophages are the primary contributor to itaconate-mediated atherosclerotic suppression, they are not the sole drivers of atherosclerosis. Endothelial cells, smooth muscle cells (SMCs), and other immune cells all play roles in atherosclerotic plaque formation and progression (72–74). Upon plaque initiation, lipids accumulate in the subendothelial region, leading to recruitment of classical monocytes and neutrophils (72, 75), where monocytes differentiate into macrophages. Macrophages are the most abundant immune cell subset in plaques, and they can also proliferate locally (41). During progression, SMCs migrate toward the developing fibrous cap, where they undergo clonal expansion (76). These cells interact with each other, both directly and indirectly, to control atherogenesis. For example, chemokines such as CCL5 induce neutrophil recruitment; meanwhile, neutrophils themselves secrete chemoattractants like CCL2 and CCL5 to attract monocytes and activate macrophages (77). Activated SMCs also secrete chemokines to promote monocyte recruitment (72). Macrophages then secrete more inflammatory cytokines and chemokines to activate and induce neutrophil and SMC migration. Activated T cells are also a substantial cell population in plaques (78) and play an overall proatherosclerotic role (79, 80). Our data support a model whereby macrophage-produced itaconate suppresses the production and secretion of proinflammatory cytokines and chemokines, which consequently dampens the migration and/or transformation of immune cells and stromal cells like SMCs and ultimately suppresses the monocyte/macrophage infiltration. Acod1 deficiency leads to increased proinflammatory polarization of macrophages, which contributes to a more inflammatory microenvironment such that other surrounding cell types likely also become more inflammatory, which in return reinforces the inflammatory profile of macrophages during atherogenesis. These different cell types form a network and collaborate to regulate atherogenesis.

There are multiple underlying mechanisms by which itaconate and its derivatives respond to inflammation, for example the IκBζ/ATF3 axis (3), succinate dehydrogenase inhibition (4), and Nrf2 activation (12). In our study, we found that Nrf2 levels increased with atherosclerosis, and that OI treatment enhanced this Nrf2 increase, whereas Acod1 deficiency caused Nrf2 levels to decrease. Subsequently, by comparing the responses of WT and *Nrf2^–/–^* BMDMs to oxLDL and/or OI in vitro and the protective effect of OI in WT and *Nrf2^–/–^* mice in vivo, we demonstrated that Nrf2 is required for itaconate-mediated suppression of atherogenesis and inflammation. Our results are consistent with a potential therapeutic role of itaconate in ocular infection, which demonstrates that itaconate exerts an antiinflammatory effect by potentiating Nrf2/HO1 signaling (51). Our results are also consistent with the recently reported mechanism through which itaconate improves donor heart preservation and function (52).

How itaconate regulates Nrf2 in atherosclerosis remains to be explored. The E3 ubiquitin ligase adaptor Kelch-like ECH-associated protein 1 (KEAP1) negatively regulates Nrf2 (81), and itaconate was recently shown to alkylate cysteine residues on KEAP1, allowing Nrf2 accumulation and activation of its downstream target genes, including antioxidant and antiinflammatory genes (12). Itaconate may also increase oxidative phosphorylation during atherogenesis, which subsequently activates MAPK cascades that activate antioxidant response element (ARE) (82) and Nrf2 pathways (83–85). Indeed, *Acod1* knockdown significantly reduced MAPK phosphorylation in peritoneal tissue–resident macrophages in tumors (70). Furthermore, itaconate could activate Nrf2 through protein kinase R–like (PKR-like) endoplasmic reticulum kinase (PERK). Treating BMDMs with the itaconate derivative dimethyl itaconate led to increased PERK levels (3), and Nrf2 is a known direct substrate of PERK (86–88).

Itaconate and its derivatives also regulate immune responses independently of Nrf2. Dimethyl itaconate inhibits the IL-6/IκBζ axis via ATF3 independently of Nrf2 (3). Furthermore, OI inhibited NLRP3 inflammasome activation in a Nrf2-independent manner (10). In addition, endogenous itaconate was not required for particulate matter–induced Nrf2 expression or inflammatory responses (89). During particulate matter–induced inflammation, endogenous itaconate, determined through the use of *Acod1^–/–^* mice, failed to activate Nrf2 in macrophages in vitro and in vivo (89); however, Nrf2 protein levels were found to be reduced in *Acod1^–/–^* BMDMs (3, 10) and heart tissues (52) compared with WT. These results may indicate that the activation of Nrf2 by itaconate or its derivatives varies with inflammatory stimulus and the inflammatory microenvironment in vivo. Our in vitro results in BMDMs do not rule out the involvement of other signaling pathways in addition to Nrf2, which could potentially include the ATF3/IκBζ pathway (3), NLRP3-NEK7 interaction and NLRP3 inflammasome (10), and targeting GAPDH and glycolysis (47, 90). Those additional potential mechanisms by which itaconate downregulates inflammation during atherogenesis will require future investigation.

In conclusion, our study has found an important role for the TCA metabolite itaconate in downregulating inflammation and suppressing atherogenesis, at least in part via activation of the antioxidant Nrf2 pathway. Our study provides impetus for developing therapeutics that boost itaconate pathways to reduce the burden of atherosclerosis.

## Methods

Further information can be found in Supplemental Methods.

### Animals and atherosclerosis.

*Acod1^–/–^* mice were generated on the C57BL/6N background, and they were acquired from Michael Diamond’s laboratory located at Washington University (St. Louis, Missouri, USA) (20). WT C57BL/6N mice (strain 005304, Jackson Laboratory) were used as controls for *Acod1^–/–^* mice. *Acod1^fl/fl^* mice were also acquired from Michael Diamond’s laboratory but on the C57BL/6 background. Myeloid-specific *Acod1*-knockout (*Acod1^fl/fl^* *LysM*^cre^) mice were generated by crossing *Acod1^fl/fl^* mice with LysM-Cre mice (strain 004781, Jackson Laboratory) in Richard Mortensen’s laboratory at the University of Michigan. Floxed littermates (*Acod1^fl/fl^*) were used as controls for *Acod1^fl/fl^* *LysM*^cre^ mice. *Nrf2^–/–^* mice were originally purchased from Jackson Laboratory (strain 017009) and then bred and housed in animal facility at the University of Michigan. WT C57BL/6 mice (strain 000664, Jackson Laboratory) were used as controls for the *Nrf2^–/–^* mice. All mice were maintained on a 12-hour light/dark cycle with free access to food and water. All mice used in this study were 2- to 4-month-old male mice unless specifically indicated. The numbers of mice for each experiment are shown in the figure legends.

To induce atherosclerosis, we used recombinant D377Y mPCSK9-AAV8 (PCSK9-AAV) that was generated at the University of Pennsylvania Vector Core. PCSK9-AAV was diluted in sterile saline and PCSK9-AAV (5.0 × 10^9^ vector genomes/g) or vehicle was administered intraperitoneally. One week after the injection, mice were given WD (42% calories from fat; TD.88137, formerly Envigo, now Inotivco Inc) for 10 weeks to increase cholesterol level and promote atherosclerosis. The week number in Supplemental Figure 2 and Supplemental Figure 4 indicates weeks after the initiation of the WD. A standard laboratory diet (5L0D, LabDiet) was used as a control diet. In a subset of mice, OI (50 mg/kg; SML2338, Sigma-Aldrich) in 40% cyclodextrin in PBS was administered twice per week intraperitoneally to mice following the initiation of the WD. Mice were randomly assigned to treatment with OI or vehicle control.

### Histopathology.

Histology services were performed by the In Vivo Animal Core within the Unit for Laboratory Animal Medicine at the University of Michigan. Briefly, 10% formalin-fixed tissues were processed and embedded with paraffin. Tissues were then sectioned at 4μm thickness. Sectioning paradigms for mouse aortic root and BCA: aortic root, spanning approximately 250 μm, 24-μm step levels, and a total of 10 sections were collected beginning at the aortic valve leaflets; BCA, beginning at the proximal root of BCA, 100-μm step levels and a total of 10 to 15 sections were collected until the right common carotid artery/right subclavian artery junction was reached. To determine the atherosclerotic lesion size and the acellular lesion (necrotic core) area (24, 91, 92), sections were subjected to hematoxylin and eosin (H&E) staining and then traced and measured using ImageJ (NIH). A total of 10 H&E-stained sections from the aortic root and 10 to 15 H&E-stained sections from the BCA were quantified per mouse. Immunohistochemical staining was performed to detect the macrophage marker Mac2 (sc-81728, Santa Cruz Biotechnology), and nuclei were counterstained with hematoxylin. For analysis of macrophages in the aortic root, section nos. 2, 4, 6, 8, and 10 were chosen.

### Human samples.

Human coronary arteries were collected as part of an autopsy evaluation for use of the samples from a deidentified human repository for medical research. Following collection of the whole organ, coronary arteries were acquired and stored in 10% formalin indefinitely, and then transferred to 70% ethanol, and 24 hours later processed and paraffin embedded for histological analysis. The human coronary artery samples were then sectioned and stained with anti-Acod1 (ab238580, Abcam) and anti-Nrf2 (ab31163, Abcam). The occlusion level was evaluated by the autopsy report as part of the cause-of-death analysis.

### Metabolomics.

Aortas were harvested, weighed, snap-frozen in liquid nitrogen, and then kept at −80°C. Frozen aortas were homogenized with dry ice–chilled 80% methanol followed by centrifugation at 10,000*g* for 5 minutes at 4°C. The supernatant was collected and an aliquot of a volume equivalent to 10 mg of the tissue from each sample was saved at −80°C. All aliquots were dried via speed vac for mass spectrometry (MS) processing. The Agilent 1290 UHPLC system and Agilent Technologies Triple Quad 6470 Mass Spectrometer (LC-MS/MS) with a 1290 Infinity II LC Flexible Pump (Quaternary Pump), 1290 Infinity II Multisampler, and 1290 Infinity II Multicolumn Thermostat with a 6-port valve were used for metabolomics analysis. Data were collected using parameters published previously (93–95). Agilent MassHunter Workstation Software LC/MS Data Acquisition for 6400 Series Triple Quadrupole MS with version B.08.02 was used for compound optimization and sample data acquisition. Agilent MassHunter Workstation Quantitative Analysis for QQQ version 10.1, build 10.1.733.0, was used to integrate and quantitate ion abundance peak areas. Absolute itaconate concentrations were calculated using an itaconate (93598, Sigma-Aldrich) standard curve.

### scRNA-seq.

Aorta samples with atherosclerotic plaques from WT and *Acod1^–/–^* mice were analyzed by scRNA-seq using the droplet-based 10× Genomics pipeline. Single-cell suspensions from the aortic root, ascending aorta, and aortic arch, where most of the aortic plaques are, were prepared by mincing isolated tissues followed by enzymatic digestion with collagenase I (450 U/mL; LS004196, Worthington), collagenase XI (125 U/mL; C7657, Sigma-Aldrich), DNase I (60 U/mL; DN25, Sigma-Aldrich), and hyaluronidase (60 U/mL; H3506, Sigma-Aldrich) for 1 hour at 37°C with agitation (96). Suspensions were then filtered, and dead cells were removed with a Dead Cell Removal Kit (480157, BioLegend) to improve cell viability of the samples. Cells were then washed, resuspended in RPMI 1640 with 10% fetal bovine serum (FBS), and processed further by the Advanced Genomics Core at the University of Michigan. Single cells were partitioned into droplet emulsion using the Chromium Controller (10× Genomics), where cells were lysed and cDNAs were reverse transcribed and barcoded. Amplified cDNAs were used to construct a 5′ gene expression (GEX) library. All cDNA libraries were sequenced on an Illumina NovaSeq 6000 platform with 150-bp paired-end reads. Raw reads were processed by the Cell Ranger pipeline and aligned to the mm10 reference genome (version mm10-2020-A). Gene count matrices were generated and used for downstream bioinformatics analysis in the R 4.0.5 environment (https://cran.r-project.org/).

As a preprocessing step, ambient RNA contamination was removed by SoupX (97), which used empty droplets contained in the raw Cell Ranger output to calculate the profile of “soup” contamination. The corrected count matrices were then processed by the Seurat v4.1.0 package (98). Low-quality cells were filtered out with the following criteria: the number of detected genes per cell should be greater than 200, the number of unique molecule identifiers (UMIs) should be less than 50,000, the percentage of expressed mitochondrial genes smaller than 10%, and the percentage of hemoglobin genes smaller than 3%. Raw counts were normalized with the “LogNormalize” method. The top 2,000 highly variable genes were scaled and used for principal component analysis (PCA). Samples were then integrated by the Harmony package (99) using the PCA result. The first 20 dimensions of the “harmony” reduction slot were used for constructing UMAP reduction and the shared nearest neighbor graph. The FindCluster function was used to identify clusters within the graph with a resolution of 0.4. Marker genes for each cluster were identified by the FindAllMarkers function using the default Wilcoxon’s rank-sum test. Clusters were annotated using the SingleR package (100) with the mouse ImmGen data set (101) as the reference, and then confirmed manually by searching top cluster-specific marker genes within Cellmarker 2.0 (102). To improve the resolution and accuracy of cell type assignment, a step-wise hierarchical annotation approach was adopted. For example, myeloid cells were subsetted out, reintegrated by Harmony, and clustered again to annotate populations of macrophages, monocytes, neutrophils, and DCs. In a similar fashion, subpopulations of macrophages were annotated. The R package scProportionTest (103) was employed to assess differences in subpopulation abundance within macrophages, monocytes, DCs, and T cells between WT and *Acod1^–/–^*, which calculates *P* values via permutation test and confidence intervals by bootstrapping. Differences in gene expression were determined using the FindMarkers function with default parameters. Genes with adjusted *P* values of less than 0.05 and absolute values of fold change greater than 1.2 were considered differentially expressed and used for pathway enrichment analysis, which was performed using the enrichR package and the “GO_Biological_Process_2021” gene-set library (104).

To calculate Nrf2 activity score, a list of Nrf2 target genes was assembled from the literature: Hmox1, Nqo1, Pgd, Taldo1, G6pdx, Idh1, Gclm, Gclc, Gsr, Gpx1, Gpx2, Gpx3, Gpx4, Gsta1, Gsta2, Txn1, Txn2, Txnrd1, Txnip, Prdx1, Prdx2, Prdx3, Prdx4, Prdx5, Prdx6, Srxn1, Sod1, Sod2, Atf1, Ppp1r15b, Als2, Nfkbib, Nrf1, Cd36, Scarb1, Cox17, Cyp2a5, Abcc2, Abcc3, Abcc4, Akr1b3, Bcl2, Calcoco2, Areg, Cdkn2c, Fmo3, Keap1, Mcm7, Mdm2, and Park7. This gene set was used as the input for the R package UCell (57) to evaluate the Nrf2 signature scores in each cell.

### BMDMs.

BMDMs were produced by flushing bone marrow from femurs and tibias. Briefly, bone marrow cells were flushed out with ice-cold PBS. After centrifugation, cells were resuspended in RPMI+ GlutaMax medium (61870, Gibco) supplemented with 10% FBS, 30% L929-conditioned medium, and 100 U/mL penicillin and streptomycin. The cells were then cultured in a humidified incubator under 95% air and 5% CO_2_ at 37°C for 4 days. On day 4, the medium was replaced with fresh medium. Two days later, BMDMs were differentiated and ready to use. BMDMs were treated with OI (250 μM for 20 hours) alone, oxLDL (100 μg/mL for 16 hours; L34357, Thermo Fisher Scientific) alone, or OI plus oxLDL. OI was added 4 hours before oxLDL in the combined treatment. Vehicle was used as control. In another experiment, BMDMs were treated with itaconate (7.5 mM for 20 hours; 93598, Sigma-Aldrich) and/or oxLDL (400 μg/mL for 16 hours).

### Quantitative RT-PCR.

Relative mRNA expression was determined using quantitative reverse transcription polymerase chain reaction (qRT-PCR). Total RNA was extracted using TRIzol reagent (15596018, Invitrogen) and RNA was reverse transcribed to cDNA. Quantitative PCR was performed using a 7900HT fast real-time PCR system (Applied Biosystems) and relative mRNA level was analyzed using the comparative method and normalized to the internal control, *L32*. Primer sequences for qRT-PCR are shown in Supplemental Table 5.

### Western blotting.

Aortas were harvested and snap-frozen in liquid nitrogen. Protein extraction was then performed by homogenizing the frozen aortas in lysis buffer (78510, Thermo Fisher Scientific) with 1% protease inhibitor cocktail (P8340, Sigma-Aldrich) and 1% phosphatase inhibitor cocktail (P5726, Sigma-Aldrich). Tissue lysates were electrophoresed in 10% SDS-polyacrylamide gels (NP0315BOX, Invitrogen) and transferred to PVDF membranes (IB401001, Thermo Fisher Scientific). Blots were blocked in 5% BSA in PBST (1% Tween 20 in PBS) for 1 hour at room temperature or 4°C overnight. Membranes were then incubated for 1 hour at room temperature with primary antibodies against Acod1 (ab222411, Abcam), Nrf2 (12721, Cell Signaling Technology), or GAPDH (2118S, Cell Signaling Technology). After washing, membranes were incubated with secondary antibodies for 30 minutes and then illuminated with chemiluminescent substrate (34577, Thermo Fisher Scientific) using a Bio-Rad ChemiDoc.

### Multiplex assay and ELISA.

V-PLEX, U-PLEX, and R-PLEX assays from the Meso Scale Discovery multispot assay system were used to quantify proteins in aorta tissue culture medium, BMDM culture medium, and plasma. Customized panels were used according to the manufacturer’s instructions. V-PLEX Panel 1 (K15048D) includes IL-1β, IL-4, IL-6, IL-10, IL-12, and CXCL1. V-PLEX Panel 2 (K15245D) includes CCL2, CCL3, CXCL2, and CXCL10. CCL5 (K152A2K-1), TGF-β Combo (K15242K-1) and MMP9 (B22ZG-2) were measured by U-PLEX assay. TIMP1 (F22YO-3) was measured by R-PLEX assay. MMP12 was measured by ELISA (ab213878, Abcam) according to the manufacturer’s instructions.

### Statistics.

Unpaired, 2-tailed Student’s *t* test, 2-way ANOVA followed by Tukey’s post hoc test, and 2-sided Pearson’s correlation test were used for statistical analysis. In figures that used 2-way ANOVA, *P* values indicate the main effect between the indicated groups. All statistical analysis was performed in Prism (GraphPad Software). A *P* value of less than 0.05 was considered significant.

### Study approval.

Animal protocols were approved by the University of Michigan Animal Care and Use Committee. All animal procedures were performed in accordance with the NIH *Guide for Care and Use of Laboratory Animals* (National Academies Press, 2011). Human samples were collected from a deidentified human repository for medical research. As the samples were deidentified, IRB approval was not required.

### Data availability.

Raw and processed mouse scRNA-seq data have been deposited in the NCBI GEO database under the identifier GSE235749 (https://www.ncbi.nlm.nih.gov/geo/query/acc.cgi?acc=GSE235749).

All remaining data that support the findings of this study are available in the main text or the supplemental materials. The supplemental Supporting Data Values file contains numerical data for all figures.

## Author contributions

JS and YZ designed and conducted experiments, analyzed data, and wrote the manuscript. RAF, AA, SW, and PS provided resources and performed experimental work. DJT, JCD, CAL, RMM, and MS provided critical materials, guidance, and comments. DRG designed, directed, supervised the study, and wrote the manuscript. All authors reviewed the manuscript and provided final approval for submission. The order of co–first authors was based on the order in which they joined the project.

## Supplementary Material

Supplemental data

Supplemental table 1

Supplemental table 2

Supplemental table 3

Supplemental table 4

Supplemental table 5

Supporting data values

## Version 1 12/12/2023

In-Press Preview

## Version 2 02/01/2024

Electronic publication
