# Supplementary material for: Itaconate suppresses atherosclerosis by activating a Nrf2-dependent antiinflammatory response in macrophages in mice
Source: J Clin Invest. 2024 Feb 1;134(3):e173034. doi: 10.1172/JCI173034 (PMC10849764; doi:10.1172/JCI173034)
Supplement: Supplemental data [file jci-134-173034-s100.pdf]

Supplemental Materials

Itaconate suppresses atherosclerosis by activating a Nrf2-dependent anti-inflammatory response in macrophages in mice

Jianrui Song[1,†], Yanling Zhang[2,†], Ryan A. Frieler[3], Anthony Andren[3], Sherri Wood[1], Daniel J. Tyrrell[1,4], Peter Sajjakulnukit[3,5], Jane C. Deng[6,7,8], Costas A. Lyssiotis[3,8,9], Richard M. Mortensen[3,10,11], Morgan Salmon[12] and Daniel R. Goldstein[1,6,13,14]

1. Department of Internal Medicine, Division of Cardiovascular Medicine, University of Michigan, Ann Arbor, MI 48109, USA
2. Department of Biochemistry and Molecular Biology, Soochow University Medical College, Suzhou, Jiangsu 215123, China
3. Department of Molecular and Integrative Physiology, University of Michigan, Ann Arbor, MI 48109, USA
4. Department of Pathology, Heersink School of Medicine, University of Alabama at Birmingham, AL 35205, USA
5. University of Michigan Rogel Cancer Center, University of Michigan, Ann Arbor, MI 48109, USA
6. Graduate Program in Immunology, University of Michigan, Ann Arbor, MI USA
7. Department of Internal Medicine, Division of Pulmonary and Critical Care Medicine, University of Michigan, Ann Arbor, MI 48109, USA
8. Veterans Affairs Ann Arbor Healthcare System, Ann Arbor, MI 48105, USA
9. Department of Internal Medicine, Division of Gastroenterology, University of Michigan Medical School, Ann Arbor, MI 48109, USA
10. Department of Pharmacology, University of Michigan, Ann Arbor, MI 48109, USA.
11. Department of Internal Medicine, Division of Metabolism, Endocrinology, and Diabetes, University of Michigan, Ann Arbor, MI 48109, USA.
12. Department of Cardiac Surgery, University of Michigan, Ann Arbor, MI 48109, USA
13. Department of Microbiology and Immunology, University of Michigan, MI 48109, USA
14. Address for correspondence: Daniel R. Goldstein, University of Michigan, Ann Arbor, MI 48109, USA. Phone: 734-936-1193. Email: drgoldst@umich.edu

[†]These authors share the first authorship.

Supplemental Methods

Flow Cytometry

Flow cytometric analysis was performed to identify myeloid cells in atherosclerotic aorta and in blood. Single cell suspension from aorta was prepared by mincing tissues followed by enzymatic digestion with collagenase I (450 U/ml, Cat# LS004196, Worthington), collagenase XI (125 U/ml, Cat# C7657, Sigma-Aldrich), DNase I (60 U/ml, Cat# DN25, Sigma-Aldrich) and hyaluronidase (60 U/ml, Cat# H3506, Sigma-Aldrich) for 1 h at 37°C with agitation(1). Single cell suspension from blood was prepared after red blood cell lysis. Aqua staining (L34966A, ThermoFisher Scientific) was used to distinguish between live and dead cells. Cells were then incubated with the following antibodies: CD45 FITC (103108, Biolegend), F4/80 PE (123110, Biolegend), CD11b BV421 (101236, Biolegend), Ly6C PerCP (128028, Biolegend) and Ly6G APC (127614, Biolegend). Flow cytometry was performed using a Bio-Rad ZE5 flow cytometer and analyzed with FlowJo V10.8 software. Cell sorting was performed using a Sony MA900. Macrophages were identified as $CD45^+CD11b^+F4/80^+$; neutrophils were identified as $CD45^+CD11b^+Ly6G^+$; monocytes were identified as $CD45^+CD11b^+Ly6C^+Ly6G^-$ and monocytes were further divided into $Ly6C^{low}$ and $Ly6C^{high}$ populations based on the expression of Ly6C. Absolute cell numbers were normalized to tissue weight for analysis. Cellular ROS production was measured by CellROX® Deep Red Flow Cytometry Assay Kit (C10491, ThermoFisher Scientific) according to the manufacturer's instructions.

Cholesterol Assay

Fasting plasma was collected at indicated time points by tail bleeding. Cholesterol levels were determined by colorimetric assay (STA-384, Cell Biolabs).

Glucose/Insulin Tolerance Test (GTT/ITT) and Insulin Assay

Glucose and insulin tolerance tests were performed 6 h after food withdrawal. Mice were then injected intraperitoneally with glucose (1.75-2.25 g/kg lean mass) or insulin (1.5-2 U/kg lean mass). A glucometer was used to measure glucose in the tail vein blood at 0, 15, 30, 45, 60, 90 and 120 min after injection. Lean mass was measured by EchoMRI (Echo Medical Systems, Houston, TX USA). Plasma insulin concentrations before and during glucose challenge were determined with an ultrasensitive insulin ELISA kit (90080, Crystal Chem).

Metabolism assay

Real-time measurements of oxygen consumption rate (OCR) and extracellular acidification rate (ECAR) were performed using a Seahorse XFe96 Analyzer (Agilent). Macrophages were isolated from peritoneal cavity or sorted from aorta. Approximately 400,000 peritoneal macrophages or 100,000 sorted macrophages were seeded into Seahorse XFe96 cell culture microplates. Each biological replicate of sorted macrophages was pooled from 2 atherosclerotic or 3 non-atherosclerotic mice.
1 h before analysis, the cells were changed into either Seahorse assay medium (103680100, Agilent) supplemented with 1 mM pyruvate, 2 mM glutamine and 10 mM glucose for mitochondrial stress test or Seahorse assay medium supplemented with 2

mM glutamine for glycolysis stress test. Mitochondrial (103015-100, Agilent) and glycolysis (103020-100, Agilent) stress tests were performed according to the manufacturer's instructions. For the mitochondrial stress test, the injections were: 1.5 μM oligomycin, 3 μM FCCP and 0.5 μM of both rotenone and antimycin A. OCR reads were taken from mitochondrial stress tests and ECAR reads were taken from glycolysis stress tests.

Supplemental Figures

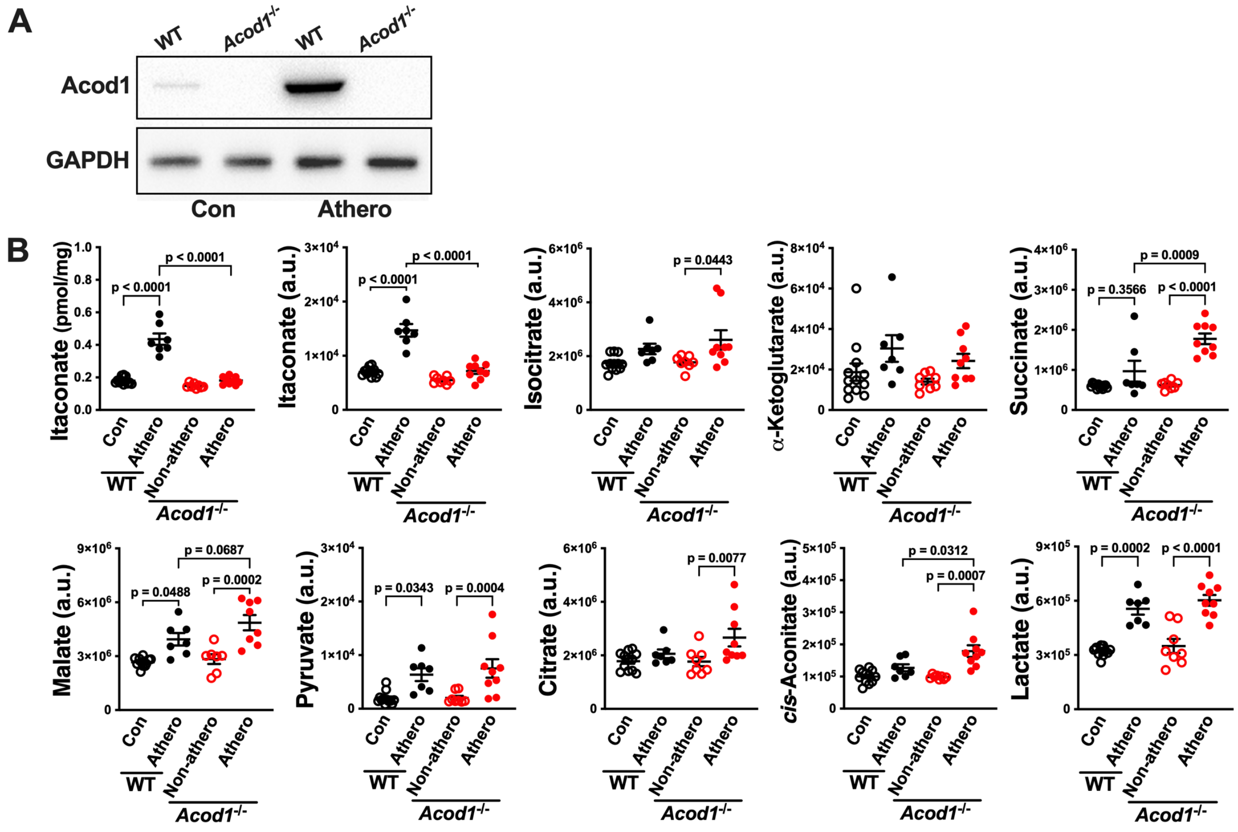

Supplemental Figure 1. Itaconate production decreases in *Acod1*$^{-/-}$ aorta. (A) Aorta lysates from WT and *Acod1*$^{-/-}$ mice with or without atherosclerosis were separated by gel electrophoresis, and proteins were detected by western blot with indicated antibodies. **(B)** Atherosclerosis in WT and *Acod1*$^{-/-}$ mice was induced via AAV-PCSK9 injection and a western-diet (WD) feeding for 10 weeks. Relative metabolite abundance by metabolomics in non-atherosclerotic WT (Con, n=12) and *Acod1*$^{-/-}$ (n=8) and atherosclerotic WT (n=7) and *Acod1*$^{-/-}$ (n=9) aortas was measured, including itaconate, isocitrate, α-ketoglutarate, succinate, malate, pyruvate, citrate, *cis*-aconitate and lactate. a.u., arbitrary units based on mass spectrometry peak area. The absolute concentrations of itaconate were also determined. Data presented here share the same data about WT mice with Figure 1C. Results are presented as means ± SEM. 2-way ANOVA followed by Tukey's post hoc test was used for statistical analysis.

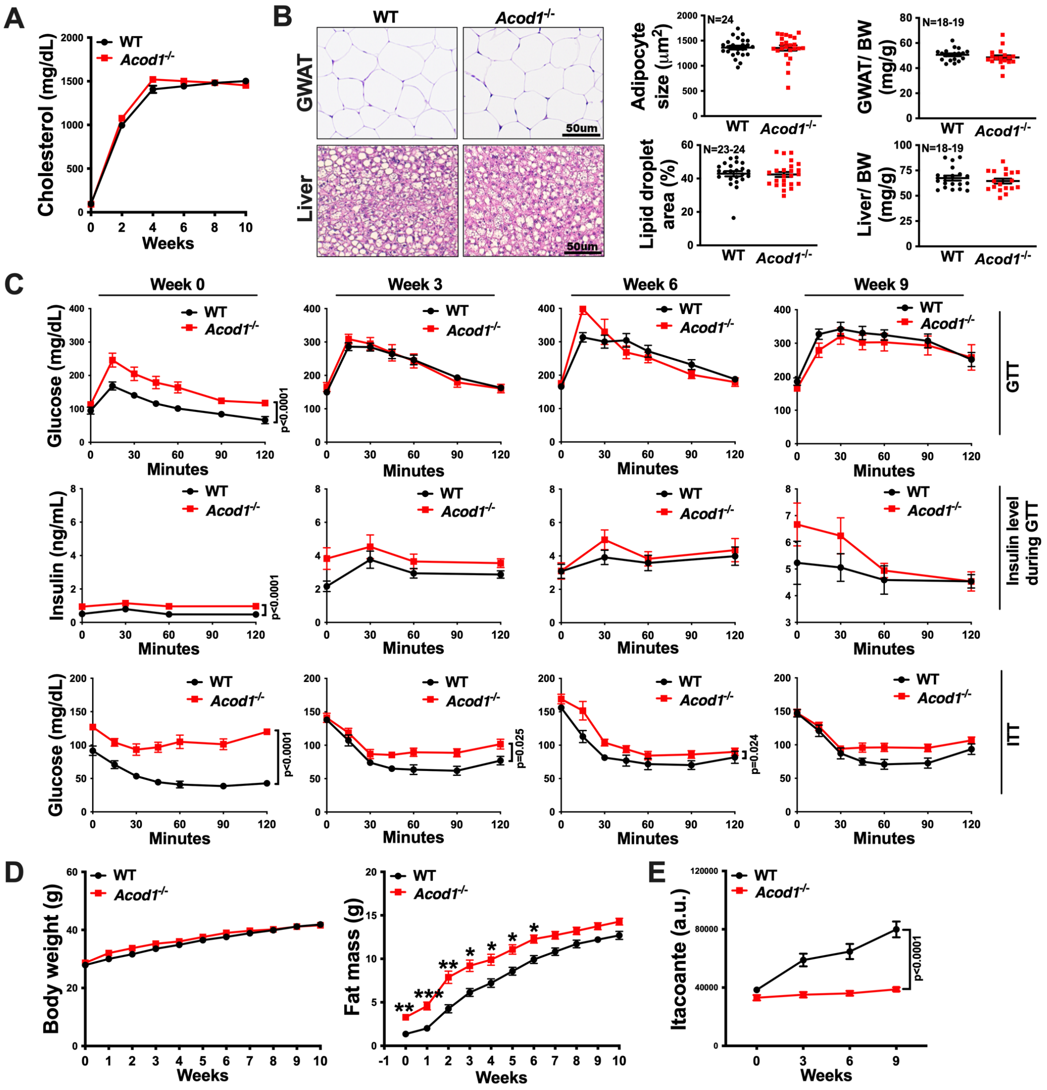

Supplemental Figure 2. Lipid and glucose metabolism in WT and *Acod1*$^{-/-}$ mice. (A) Fasting plasma was collected at indicated time points from WT (n=12) and *Acod1*$^{-/-}$ (n=11) mice during the development of atherosclerosis, and then fasting cholesterol was measured accordingly. **(B)** Representative images of H&E-stained sections of gonadal white adipose tissue (GWAT) and liver from atherosclerotic WT and *Acod1*$^{-/-}$ mice. The quantifications of adipocyte size in GWAT (n=24/group), the percentage of lipid droplet areas in liver (n=23-24/group), GWAT weight to body weight (BW) ratio (n=18-19/group) and liver weight to BW ratio (n=18-19/group) are shown on the right. **(C)** Glucose tolerance test (GTT) and insulin tolerance test (ITT) were performed in WT and *Acod1*$^{-/-}$ mice at the indicated time points during the development of atherosclerosis. Insulin secretion was also determined before and after glucose challenge (n=6-7/group). **(D)** The body weight (n=20) and fat mass (n=12-13) of WT and *Acod1*$^{-/-}$ mice during their development of atherosclerosis were measured by body composition analyzer, EchoMRI. *** p<0.005; ** p<0.01; * p<0.05. **(E)** Relative metabolomic analysis of itaconate in aortas of WT (n=6) and *Acod1*$^{-/-}$ (n=5) mice during their development of atherosclerosis. a.u., arbitrary units based on mass spectrometry peak area (not directly comparable between experiments). Results are presented as means ± SEM. 2-way ANOVA was used in **(A)** and **(C-E)** and unpaired two-tailed Student's t-test was used in **(B)** for statistical analysis.

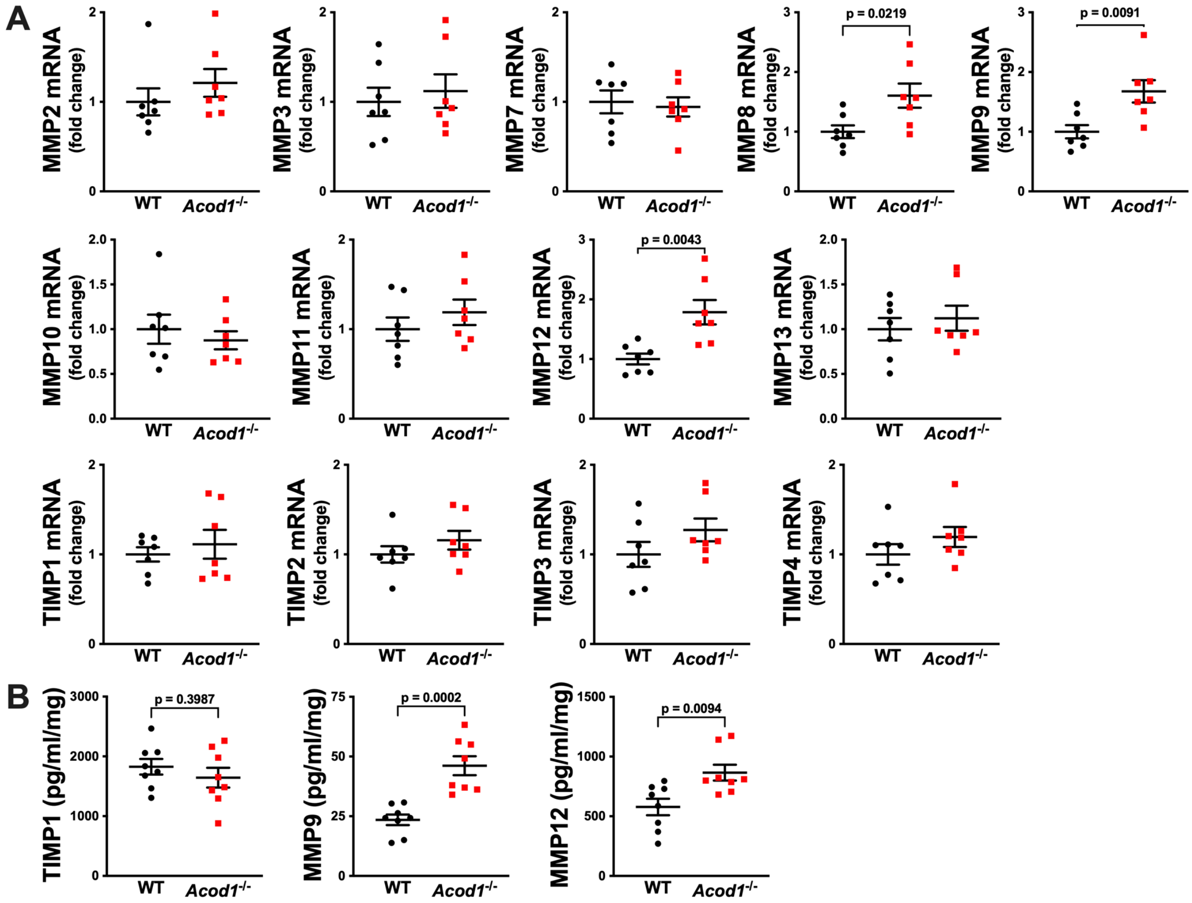

Supplemental Figure 3. Itaconate regulates mediators involved in tissue repair. **(A)** The mRNA levels of genes involved in tissue repair (MMP2, MMP3, MMP7, MMP8, MMP9, MMP10, MMP11, MMP12, MMP13, TIMP1, TIMP2, TIMP3 and TIMP4) in atherosclerotic aortas from WT and *Acod1*$^{-/-}$ mice were measured by qRT-PCR (n=7/group). **(B)** Protein levels of TIMP1 and MMP9 were measured by multiplex assay and, separately, protein levels of MMP12 were measured by ELISA in tissue culture medium of aortas from atherosclerotic WT and *Acod1*$^{-/-}$ mice (n=8/group). Results are presented as means ± SEM. Unpaired two-tailed Student's t-test was used for statistical analysis.

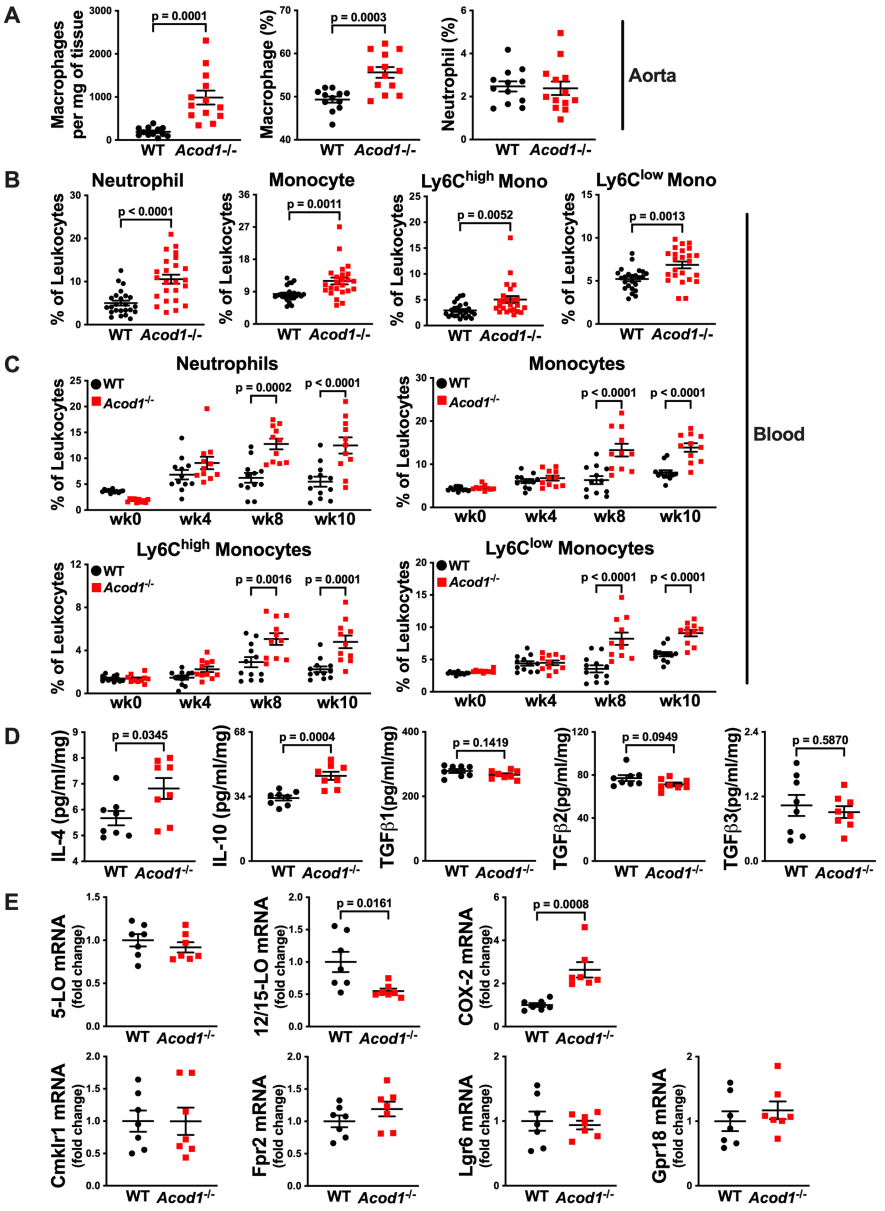

Supplemental Figure 4. Acod1 deficiency increases local and systemic inflammation.
(A) Flow cytometric analysis of absolute macrophage numbers and the percentage of macrophages and neutrophils in total leukocytes in aorta of atherosclerotic WT (n=12) and *Acod1*$^{-/-}$ (n=13) mice. Macrophage numbers are expressed per milligram aorta tissue. **(B)** The percentage of neutrophils, total monocytes and the subsets of Ly6C^{high} and Ly6C^{low} monocytes in total circulating leukocytes of atherosclerotic WT and *Acod1*$^{-/-}$ mice were determined by flow cytometry (n=24/group) at the end of the 10-week treatment. **(C)** The percentage of neutrophils, monocytes, Ly6C^{high} and Ly6C^{low} monocyte subsets in total circulating leukocytes of WT (n=12) and *Acod1*$^{-/-}$ (n=11) mice during the development of atherosclerosis for 10 weeks. **(D)** Protein levels of IL-4, IL-10, TGFβ1, TGFβ2 and TGFβ3 in supernatant of cultured aortas from atherosclerotic WT and *Acod1*$^{-/-}$ mice were measured by multiplex assay (n=8/group). **(E)** The mRNA levels of genes involved in inflammation resolution (5-LO, 12/15-LO, COX-2, Cmklr1, Fpr2, Lgr6 and Gpr18) in atherosclerotic aortas from WT and *Acod1*$^{-/-}$ mice were measured by qRT-PCR (n=7/group). Results are presented as means ± SEM. Unpaired two-tailed Student's t-test was used in **(A-B)** and **(D-E)** and 2-way ANOVA was used in **(C)** for statistical analysis.

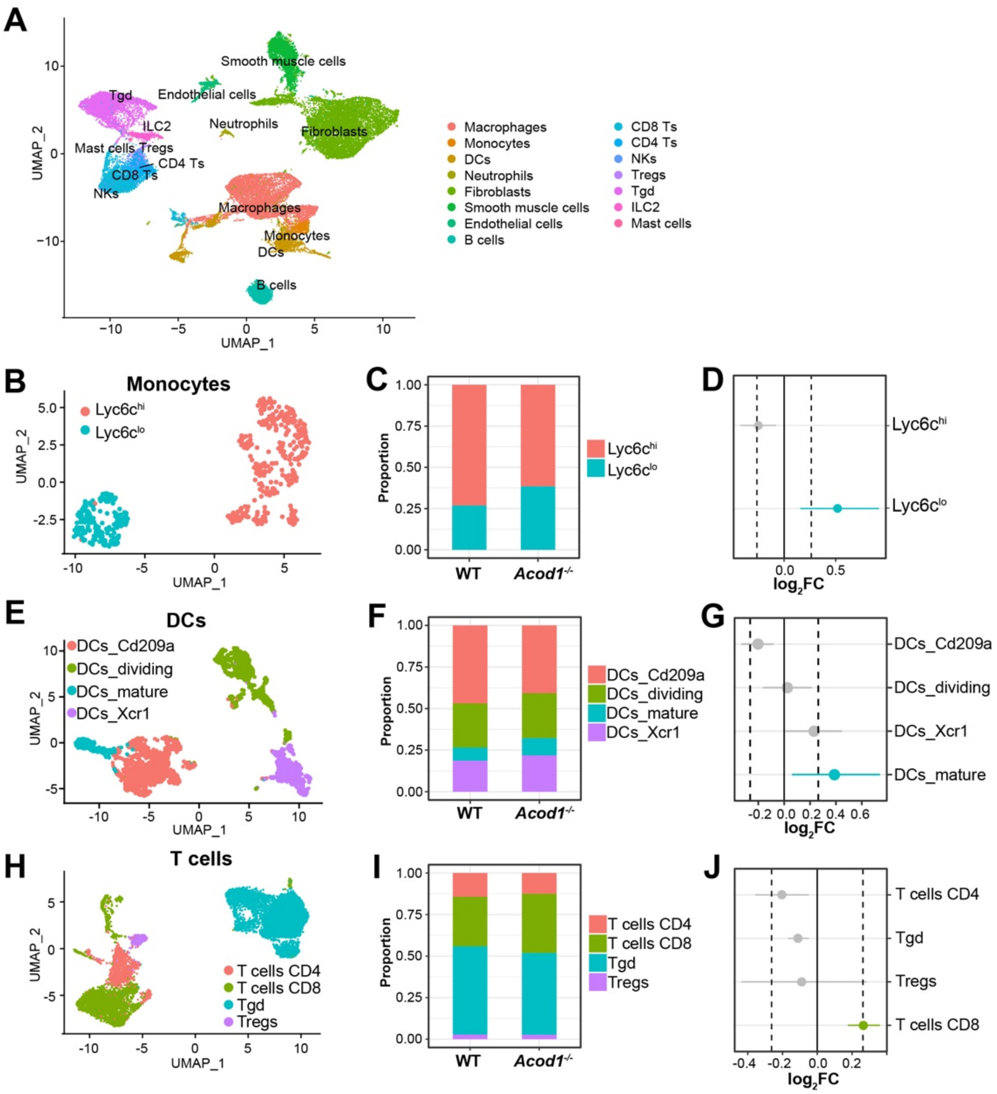

Supplemental Figure 5. Single cell analysis of other immune cells in plaque-containing atherosclerotic aorta in WT and *Acod1*$^{-/-}$ mice. (A) UMAP plot showing all cell types revealed by single cell RNA sequencing. **(B)** UMAP plot showing two subpopulations of monocytes: Ly6c^{high} (high in Ly6c, Ccr2 and Cd14) and Ly6c^{low} (low in Ly6c, high in Ace, Ear2, and Itgal). **(C)** The proportion of monocyte subpopulations from atherosclerotic WT and *Acod1*$^{-/-}$ aortas. **(D)** Differential abundance testing of changes in the proportion of monocyte subpopulations in atherosclerotic *Acod1*$^{-/-}$ aortas. **(E)** UMAP plot showing four subpopulations of dendritic cells (DCs): Cd209^{+} DCs (high in Cd209 and MHC II), Xcr1^{+} DCs (high in Xcr1 and Itgae), mature/migratory DCs (high in Ccr7, Cd83, Fscn1, Cacnb3), and dividing DCs (high in Cdk1 and histones). **(F)** The proportion of DC subpopulations from atherosclerotic WT and *Acod1*$^{-/-}$ aortas. **(G)** Differential abundance testing of changes in the proportion of DC subpopulations in atherosclerotic *Acod1*$^{-/-}$ aortas. **(H)** UMAP plot showing four subpopulations of T cells: CD4^{+} T cells (high in Cd4, Slamf6, Cd40lg), CD8^{+} T cells (high in Cd8a and Gzmk), Tgd (high in Trdc), and Tregs (high in Foxp3). **(I)** The proportion of T cell subpopulations from atherosclerotic WT and *Acod1*$^{-/-}$ aortas. **(J)** Differential abundance testing of changes in the proportion of T cell subpopulations in atherosclerotic *Acod1*$^{-/-}$ aortas. In **(D)**, **(G)** and **(J)**, clusters that passed the threshold of adjusted p values < 0.05 and FC > 1.2 were deemed significant and colored. FC, fold change.

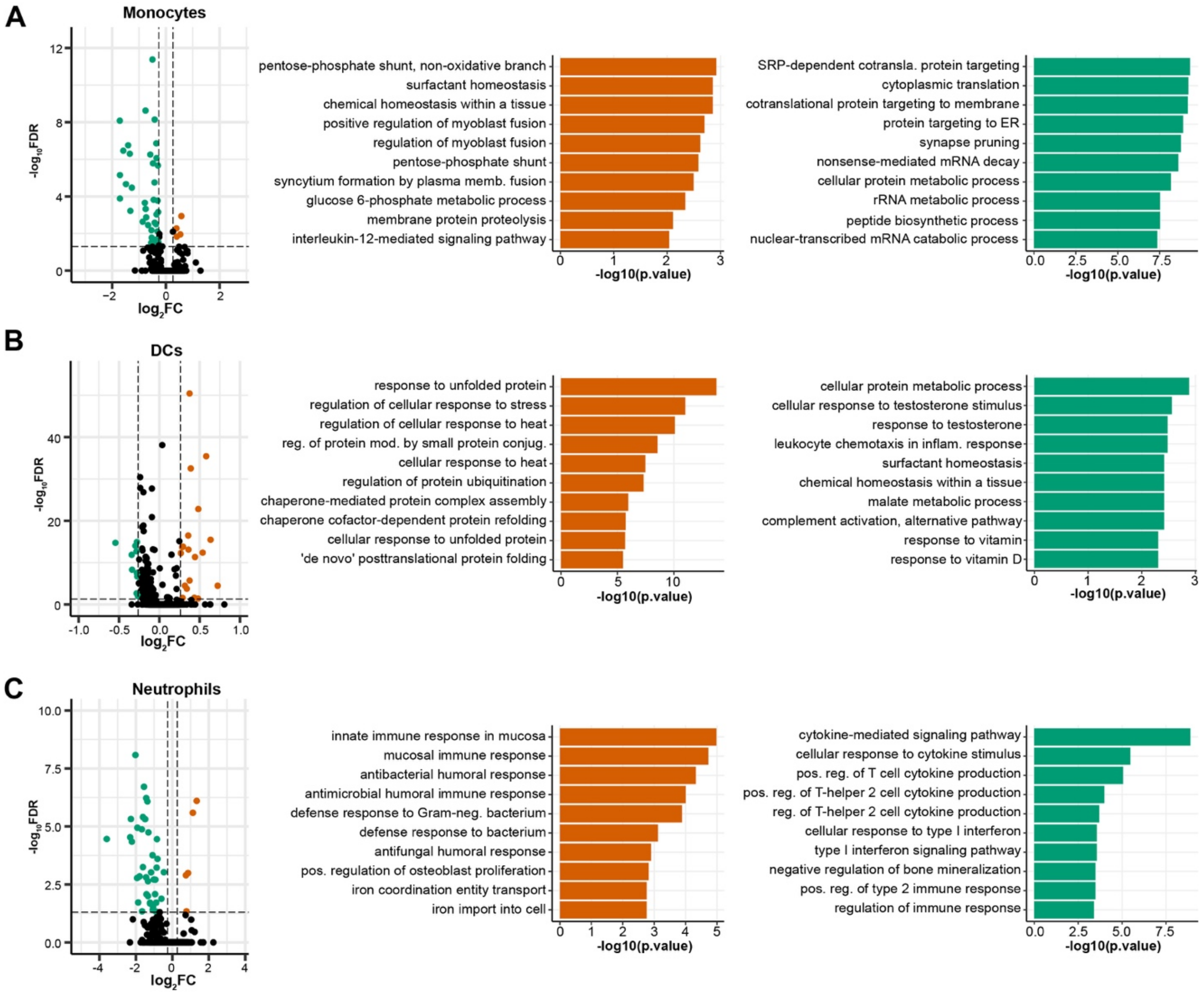

Supplemental Figure 6. Analysis of differentially expressed genes in monocytes, DCs and neutrophils in the single cell RNAseq dataset of atherosclerotic aorta of WT and *Acod1*$^{-/-}$ mice. (A) Volcano plot showing differentially expressed genes in monocytes from atherosclerotic *Acod1*$^{-/-}$ aortas. Up- and down-regulated genes were colored orange and green, respectively. FDR, false discovery rate. Right: Gene ontology analysis of up-regulated (orange) and down-regulated (green) genes in monocytes from atherosclerotic *Acod1*$^{-/-}$ aortas. **(B)** Volcano plot and gene ontology analysis of DCs. **(C)** Volcano plot and gene ontology analysis of neutrophils.

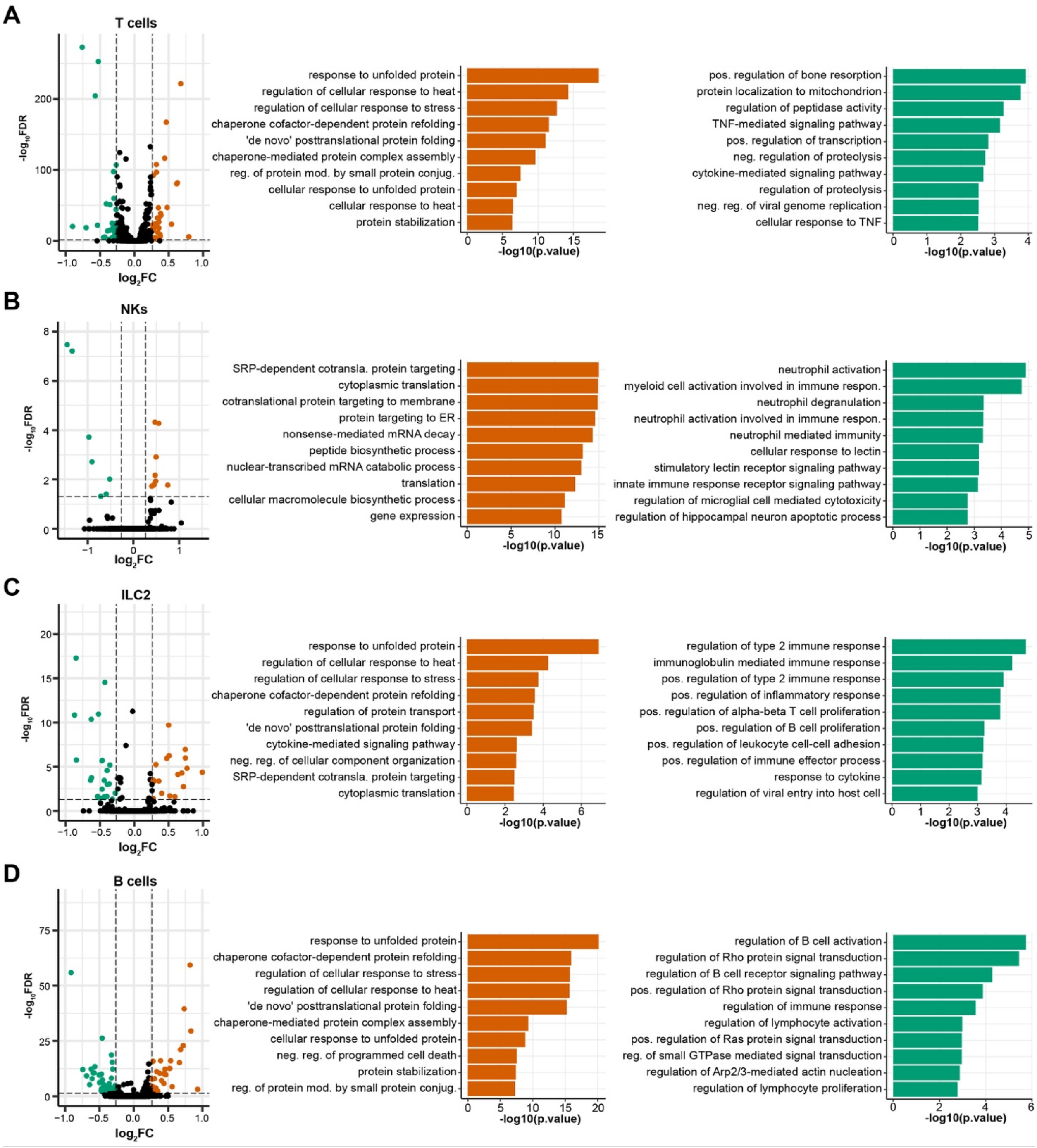

Supplemental Figure 7. Analysis of differentially expressed genes in T cells, NK cells, ILC2 cells and B cells in the single cell RNAseq dataset of atherosclerotic aorta of WT and *Acod1*$^{-/-}$ mice. **(A)** Volcano plot showing differentially expressed genes in T cells from atherosclerotic *Acod1*$^{-/-}$ aortas. Up- and down-regulated genes were colored orange and green, respectively. FDR, false discovery rate. Right: Gene ontology analysis of up-regulated (orange) and down-regulated (green) genes in T cells from atherosclerotic *Acod1*$^{-/-}$ aortas. **(B)** Volcano plot and gene ontology analysis of NK cells. **(C)** Volcano plot and gene ontology analysis of ILC2 cells. **(D)** Volcano plot and gene ontology analysis of B cells.

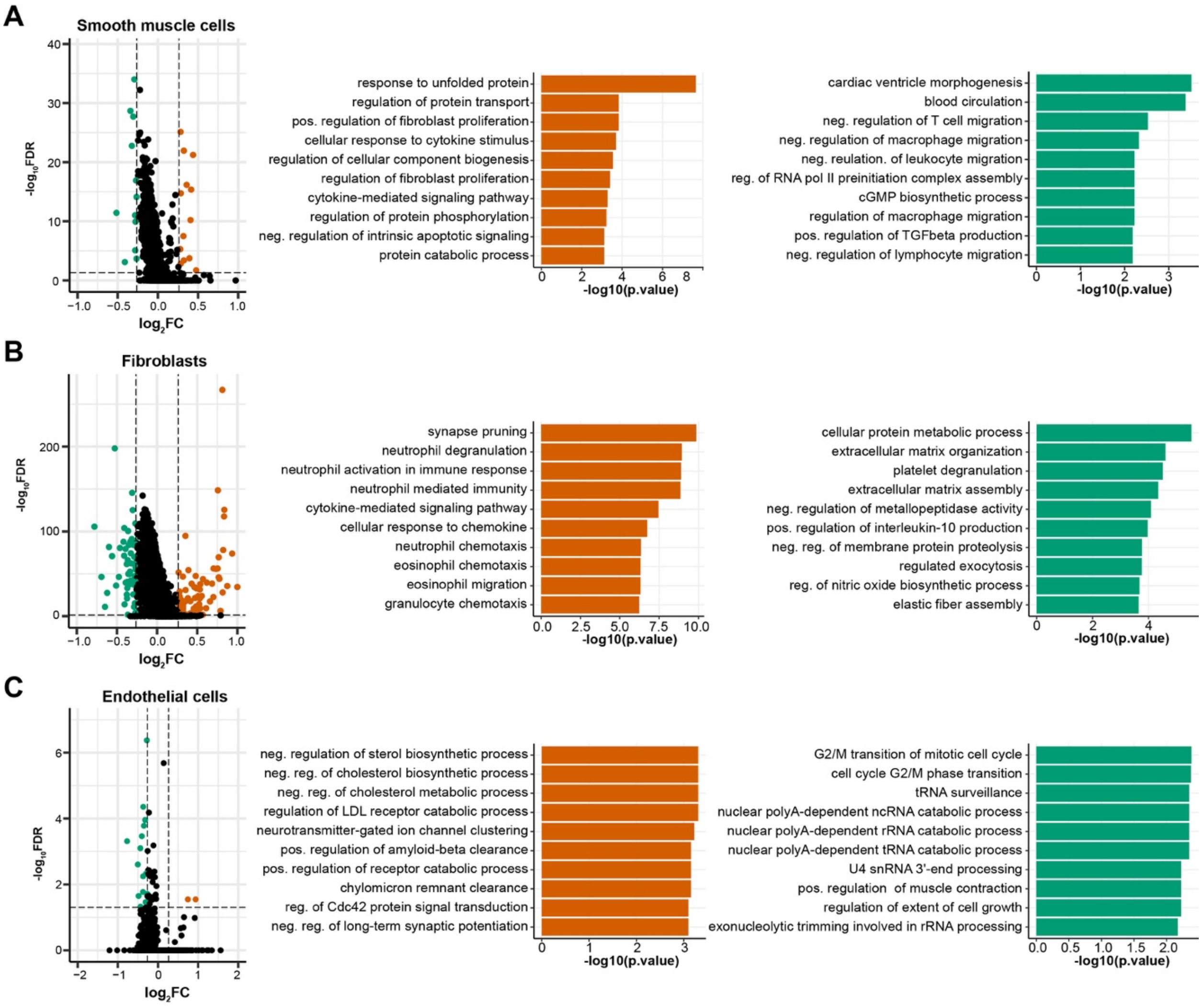

Supplemental Figure 8. Analysis of differentially expressed genes in smooth muscle cells, fibroblasts and endothelial cells in the single cell RNAseq dataset of atherosclerotic aorta of WT and *Acod1*$^{-/-}$ mice. (A) Volcano plot showing differentially expressed genes in smooth muscle cells from atherosclerotic *Acod1*$^{-/-}$ aortas. Up- and down-regulated genes were colored orange and green, respectively. FDR, false discovery rate. Right: Gene ontology analysis of up-regulated (orange) and down-regulated (green) genes in smooth muscle cells from atherosclerotic *Acod1*$^{-/-}$ aortas. **(B)** Volcano plot and gene ontology analysis of fibroblasts. **(C)** Volcano plot and gene ontology analysis of endothelial cells.

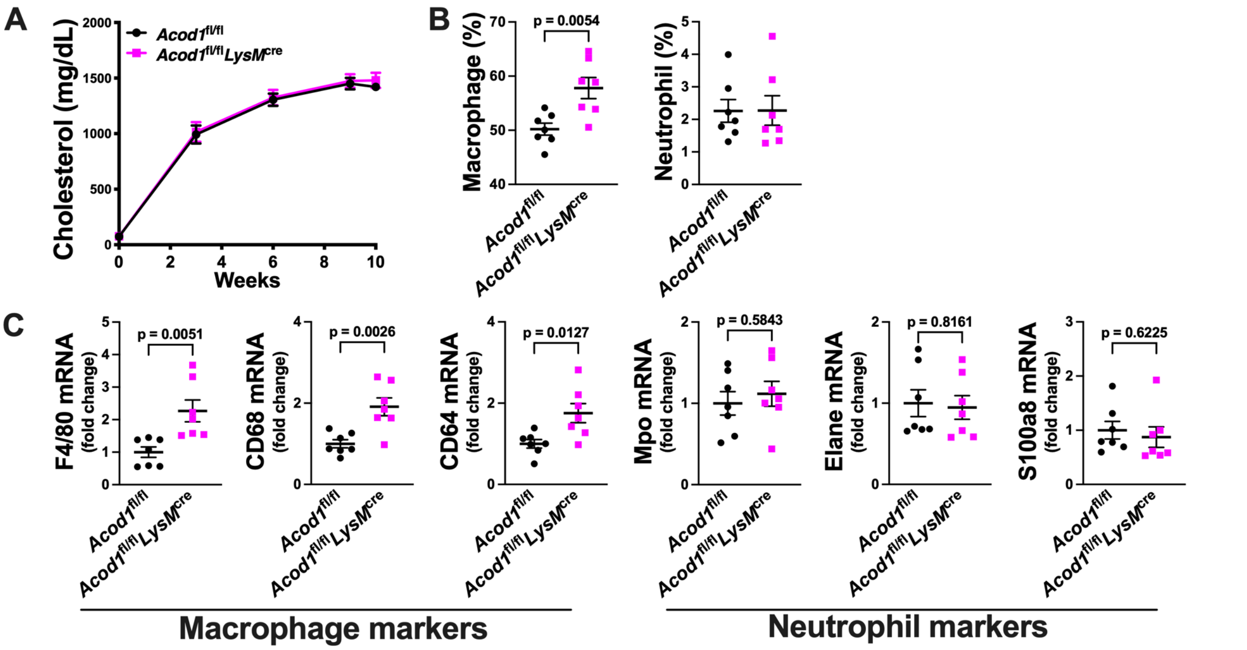

Supplemental Figure 9. Itaconate is less involved in neutrophil populations in atherosclerotic aorta. (A) Fasting cholesterol was measured in plasma of *Acod1*$^{fl/fl}$ (n=11) and *Acod1*$^{fl/fl}$*LysM*^{cre} (n=12) mice at indicated time points during the development of atherosclerosis. **(B)** Flow cytometric analysis of the percentage of macrophages and neutrophils in total leukocytes from aorta of atherosclerotic *Acod1*$^{fl/fl}$ and *Acod1*$^{fl/fl}$*LysM*^{cre} mice (n=7/group). **(C)** Gene expression of macrophage markers (F4/80, CD68 and CD64) and neutrophil markers (Mpo, Elane and S100a8) in aortas of atherosclerotic *Acod1*$^{fl/fl}$ and *Acod1*$^{fl/fl}$*LysM*^{cre} mice (n=7/group) was measured by qRT-PCR. Results are presented as means ± SEM. 2-way ANOVA was used in **(A)** and unpaired two-tailed Student's t-test was used in **(B-C)** for statistical analysis.

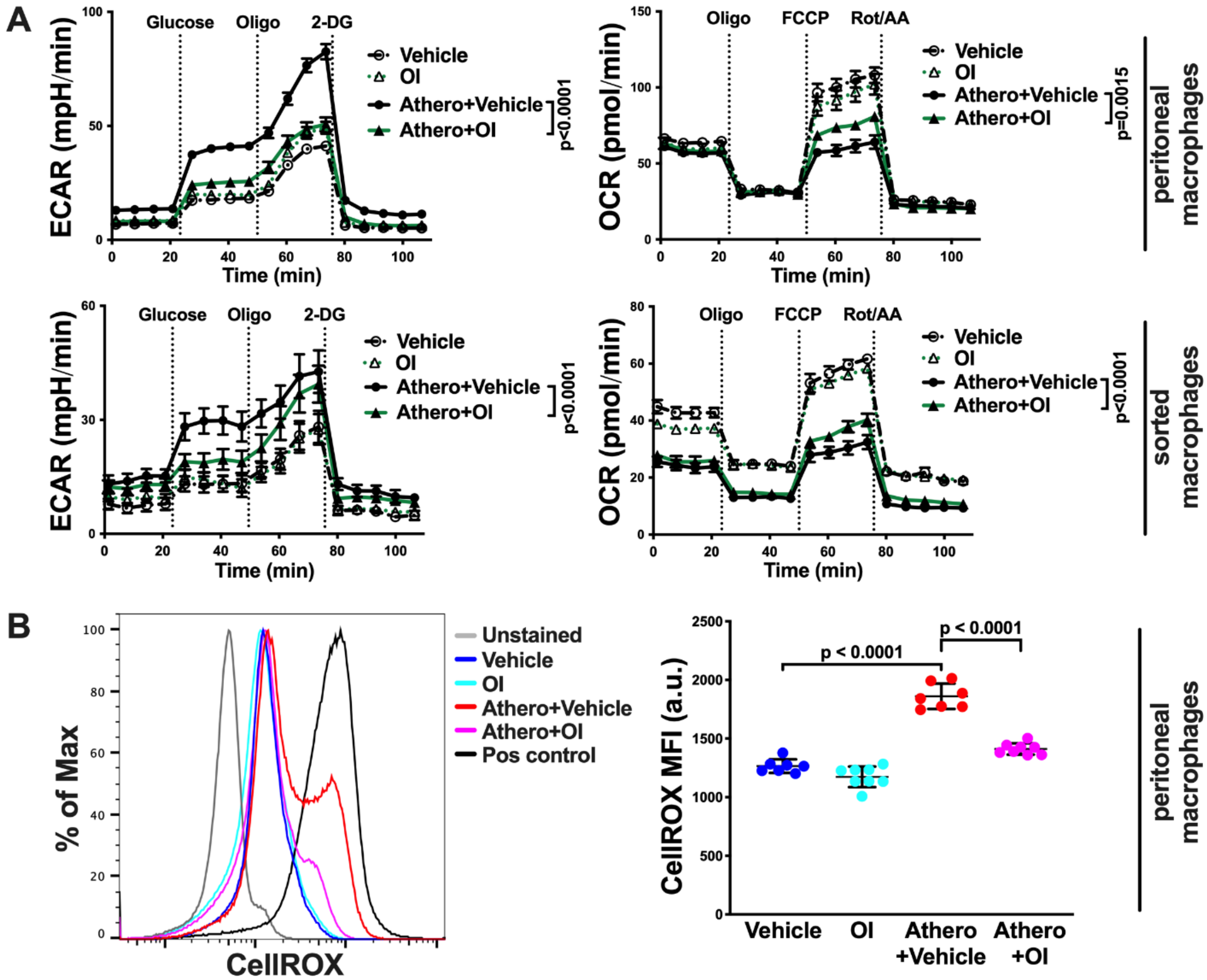

Supplemental Figure 10. 4-octyl itaconate decreases glycolysis and ROS production in macrophages from atherosclerotic mice. Macrophages were isolated from peritoneal cavity or sorted from aorta of non-atherosclerotic and atherosclerotic mice with or without 4-octyl itaconate (OI) administration. **(A)** Real-time extracellular oxygen consumption rate (OCR) and extracellular acidification rate (ECAR) measurements in macrophages from mice with indicated treatment (n=8-16/group). Vehicle: vehicle control; OI: 4-octyl itaconate; Athero+Vehicle: atherosclerosis and vehicle; Athero+OI: atherosclerosis and 4-octyl itaconate. **(B)** Histograms of ROS production in peritoneal macrophages from indicated mice are shown, and the quantification of CellROX mean fluorescence intensity (MFI) is on the right (n=7-8/group). Peritoneal macrophages (from non-atherosclerotic mice) exposed to LPS (1000ng/ml) for 12hs were used as positive control (Pos control). a.u., arbitrary units. Results are presented as means ± SEM. 2-way ANOVA was used for statistical analysis.

Supplemental Figure 11

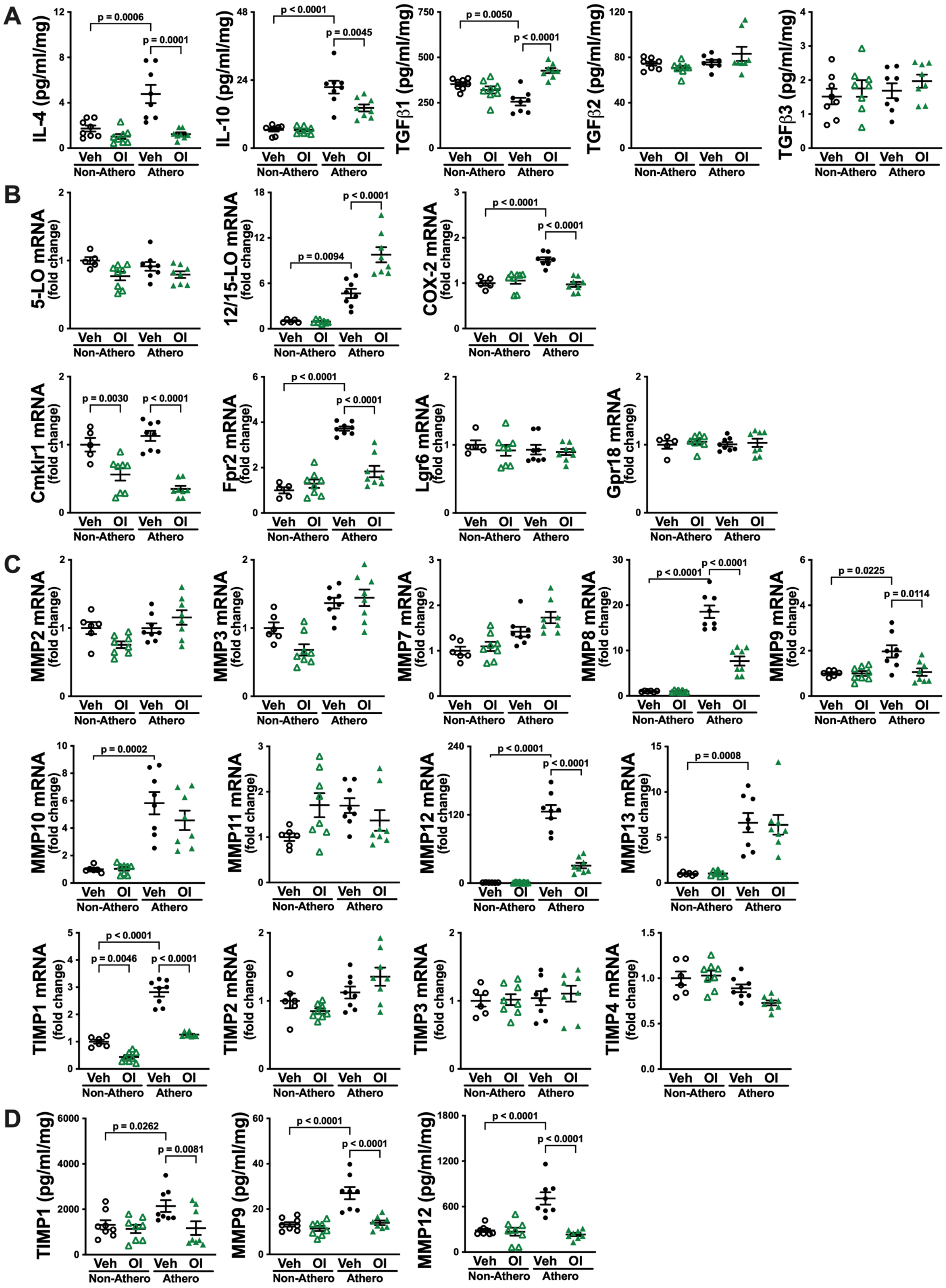

Supplemental Figure 11. 4-octyl itaconate regulates cytokines and mediators involved in inflammation resolution and tissue repair. WT mice were subjected to the following treatments: 4-octyl itaconate (OI) only, atherosclerosis (Athero) only and OI plus Athero. Vehicle was used as the control for OI, and mice without atherosclerosis (Non-Athero) were used as control mice. (**A**) Protein levels of IL-4, IL-10, TGFβ1, TGFβ2 and TGFβ3 in tissue culture medium (CM) of aortas from indicated mice were measured by multiplex assay (n=8/group). **(B-C)** The mRNA level of genes involved in inflammation resolution, like 5-LO, 12/15-LO, COX-2, Cmklr1, Fpr2, Lgr6 and Gpr18, and tissue repair including MMP2, MMP3, MMP7, MMP8, MMP9, MMP10, MMP11, MMP12, MMP13, TIMP1, TIMP2, TIMP3 and TIMP4, were measured in aorta tissues of indicated mice by qRT-PCR (n=6-8/group). (**D**) Protein levels of TIMP1 and MMP9 were measured by multiplex assay and, separately, protein level of MMP12 was measured by ELISA in aorta CM from indicated mice (n=8/group). Protein levels in aorta CM were normalized to tissue weight for analysis. Results are presented as means ± SEM. 2-way ANOVA followed by Tukey's post hoc test was used for statistical analysis.

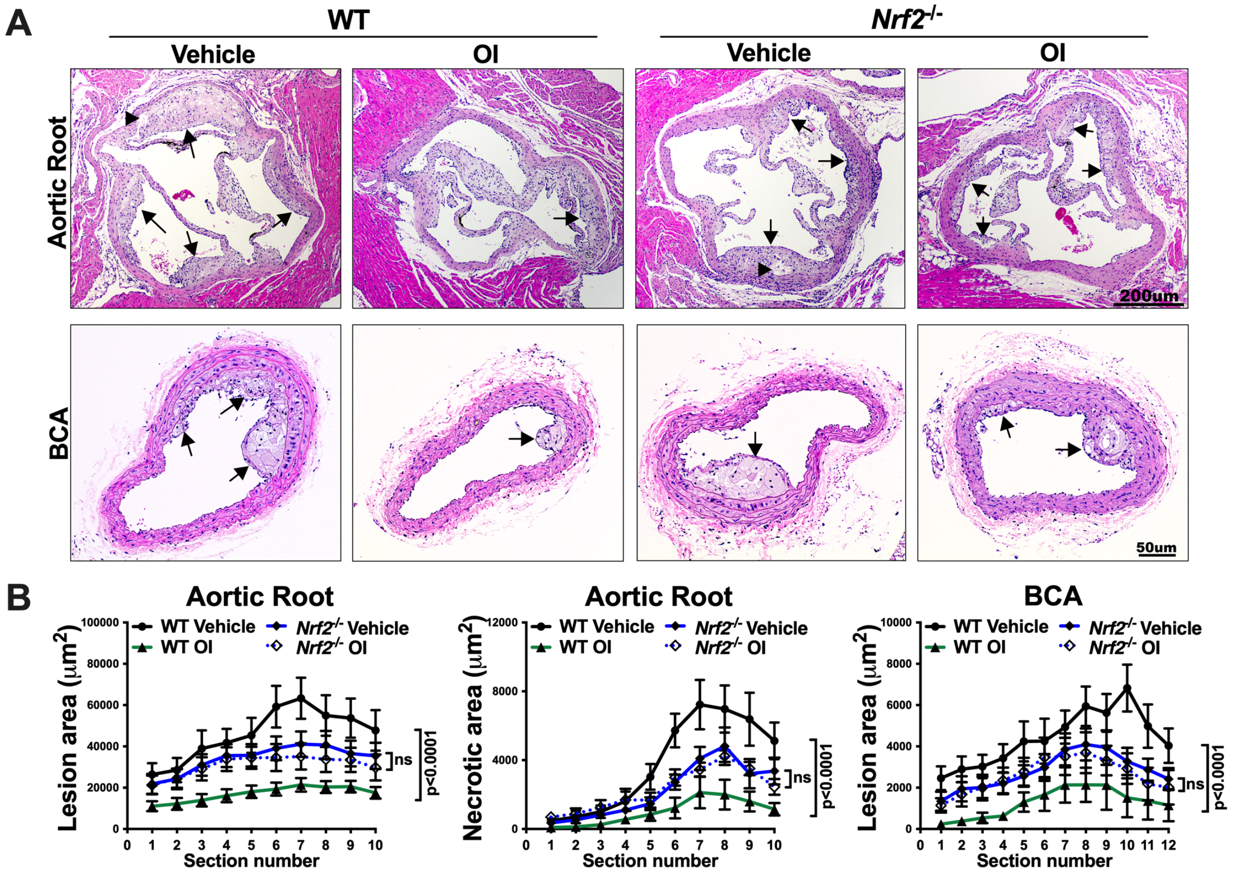

Supplemental Figure 12. The suppression of atherogenesis by itaconate derivative 4-octyl itaconate is Nrf2-dependent. Atherosclerosis was induced in both WT and *Nrf2*$^{-/-}$ mice via PCSK9-AAV administration followed by 10-week western diet. **(A)** Representative images of H&E-stained aortic root and brachiocephalic artery (BCA) sections of indicated atherosclerotic mice. WT Vehicle: atherosclerotic WT mice with vehicle; WT OI: atherosclerotic WT mice with 4-octyl itaconate; *Nrf2*$^{-/-}$ Vehicle: atherosclerotic *Nrf2*$^{-/-}$ mice with vehicle; *Nrf2*$^{-/-}$ OI: atherosclerotic *Nrf2*$^{-/-}$ mice with 4-octyl itaconate. Arrows indicate atherosclerotic lesions and arrowheads indicate necrotic cores. **(B)** The quantifications of lesion area and necrotic area in each section of aortic root and BCA from indicated mice are shown (n=10-16/group). Results are shown as means ± SEM. 2-way ANOVA followed by Tukey's post hoc test was used for statistical analysis.

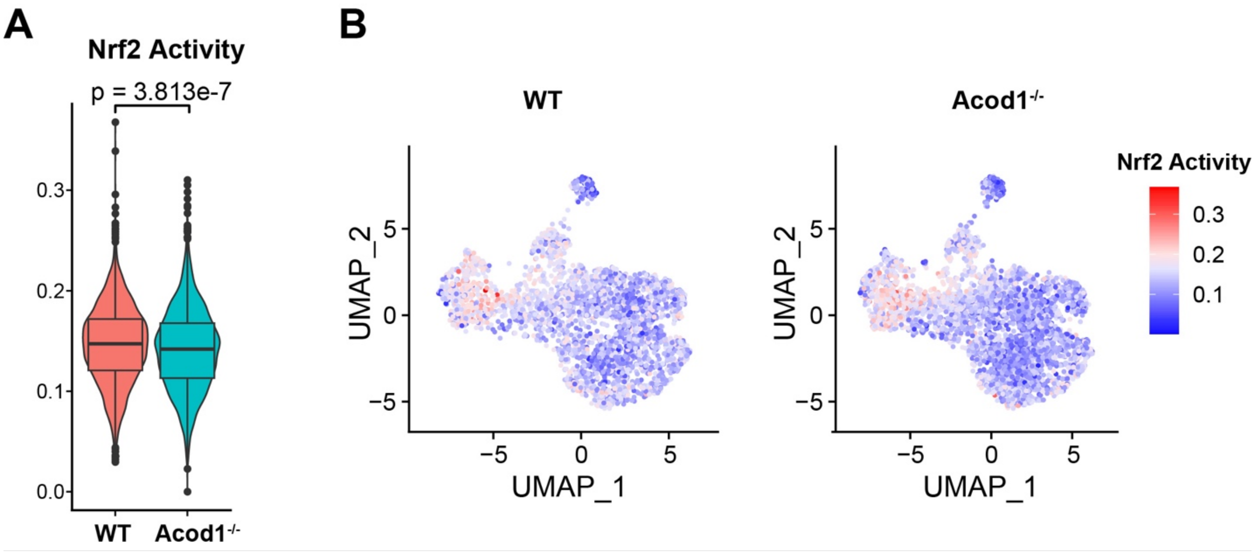

Supplemental Figure 13. Nrf2 activity in the single cell RNAseq dataset. (A) Nrf2 activity score in each macrophage from atherosclerotic plaques was calculated using the UCell R package. Scores from WT and *Acod1*$^{-/-}$ macrophages were compared by two-sided Student's t-test. **(B)** Nrf2 activity scores were overlaid on the UMAP plots of macrophages.

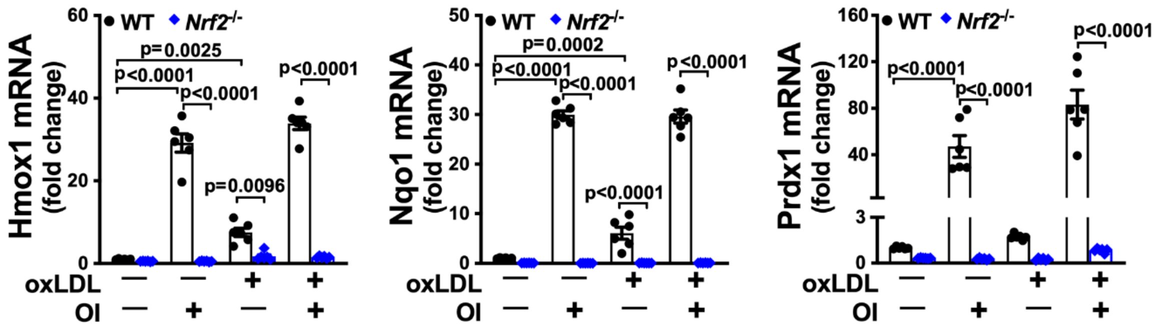

Supplemental Figure 14. Itaconate derivative 4-octyl itaconate (OI) induces the upregulation of Nrf2 targets in WT but not *Nrf2*$^{-/-}$ BMDM. Bone marrow derived macrophages (BMDMs) from WT and *Nrf2*$^{-/-}$ mice were treated with or without OI (250 μM), followed by exposure to oxLDL (100 μg/ml). Vehicle was used as control. Gene expression of Nrf2 target genes including Hmox1, Nqo1 and Prdx1 in those BMDMs was then measured by qRT-PCR (n=6/group).

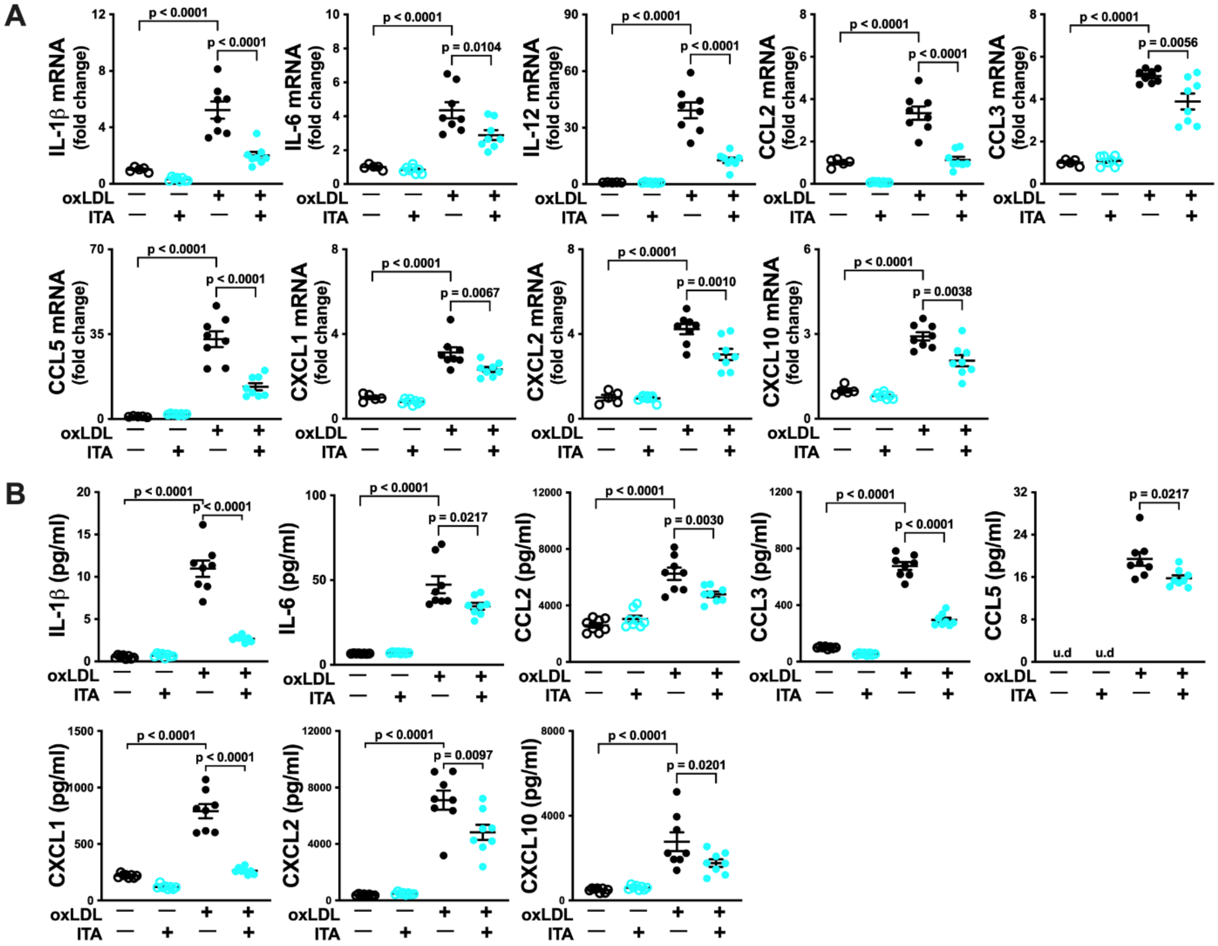

Supplemental Figure 15. Itaconate (ITA) also successfully diminishes the inflammation induced by oxLDL in BMDMs. Bone marrow derived macrophages (BMDMs) from WT mice were treated with or without ITA (7.5 mM), followed by exposure to oxLDL (400 μg/ml). Vehicle was used as control. Cells and culture medium supernatants were collected at the end of experiment, and RNA was extracted from the cells. **(A)** The mRNA levels (n=5-8/group) of the indicated cytokines and chemokines in those BMDMs were measured by qRT-PCR, and **(B)** the corresponding protein levels (n=8/group) in the cell culture supernatants of BMDMs were measured by multiplex assay, including IL-1β, IL-6, IL-12, CCL2, CCL3, CCL5, CXCL1, CXCL2 and CXCL10. u.d: undetectable. IL-12 was below detectable range in cell culture supernatant. Results are presented as means ± SEM. 2-way ANOVA followed by Tukey's post hoc test was used for statistical analysis.

Supplemental Tables

Supplemental Table 1. Marker genes for macrophage subpopulations in single cell RNAseq profiling of atherosclerosis plaques. Marker genes for each cluster were calculated with the FindAllMarkers function in the Seurat package. Wilcoxon Rank Sum test was used to compare the cluster of interest to all other clusters. Only positive markers were returned.

Supplemental Table 2. Changes in the proportion of macrophage subpopulations. The difference in subpopulation composition between WT and *Acod1*$^{-/-}$ were calculated with the scProportionTest package. Log2 fold changes, p values, adjusted p values (FDR) and confidence intervals by bootstrapping were returned.

Supplemental Table 3. Differential expression in macrophages from WT and *Acod1*$^{-/-}$ atherosclerotic plaques revealed by scRNAseq. Genes that had differential expression between WT and *Acod1*$^{-/-}$ were calculated with the FindMarkers function in the Seurat package. Wilcoxon Rank Sum test was used. The parameter logfc.threshold was set to zero to include all expressed genes. The output includes the following results: p_val, p values; avg_log2FC, log2 fold changes of the average expression of *Acod1*$^{-/-}$ cells versus WT cells; pct_1, percentage of *Acod1*$^{-/-}$ cells where the gene was detected; pct_2, percentage of WT cells where the gene was detected; p_val_adj, p values after adjustment for multiple comparison using the Bonferroni method. Differentially expressed genes (DEGs) were defined as FC > 1.2 and adjusted p values

< 0.05. The lists of upregulated and downregulated DEGs were assessed with the R package EnrichR to identify changes in GO Biological Process pathways in *Acod1*$^{-/-}$.

Supplemental Table 4. Differentially expressed genes between WT and *Acod1*$^{-/-}$ in non-macrophage cells, along with associated gene ontology pathway enrichment. Differentially expressed genes (DEGs) between WT and *Acod1*$^{-/-}$ were calculated in monocytes, DCs, neutrophils, T cells, NK cells, ILC2 cells, B cells, smooth muscle cells (SMCs), fibroblasts, and endothelial cells using the FindMarkers() function from the Seurat package. DEGs were defined as in Table S3. Pathway analysis on upregulated and downregulated genes was performed as in Table S3.

Supplemental Table 5. Sequences of primers used in this study. The primers were synthesized by Integrated DNA Technologies. L32, 60S ribosomal protein L32, was used as an internal control.

References

1. Hu D, Yin C, Mohanta SK, Weber C, and Habenicht AJ. Preparation of Single Cell Suspensions from Mouse Aorta. *Bio Protoc.* 2016;6(11).
