# Supplementary material for: Itaconate suppresses atherosclerosis by activating a Nrf2-dependent antiinflammatory response in macrophages in mice
Source: J Clin Invest. 2024 Feb 1;134(3):e173034. doi: 10.1172/JCI173034 (PMC10849764; doi:10.1172/JCI173034)
Supplement: Supplemental table 5 [file jci-134-173034-s105.pdf]

Supplemental Table. 5

Gene	Forward	Reverse
Acod1	CCAAAGAGATTCCACCCTCCC	CCTGCGTGGGAAACAGCAAT
IL-1β	AAGAGCTTCAGGCAGGCAGTATCA	TGCAGCTGTCTAATGGGAACGTCA
IL-6	GAGGATACCACTCCCAACAGACC	AAGTGCATCATCGTTGTTCATACA
IL-12	TGGTTTGCCATCGTTTTGCTG	ACAGGTGAGGTTCACTGTTTCT
CCL2	TCACCTGCTGCTACTCATTCACCA	TACAGCTTCTTTGGGACACCTGCT
CCL3	TTCTCTGTACCATGACACTCTGC	CGTGGAATCTTCCGGCTGTAG
CCL5	TCGTGCCCACGTCAAGGAGTATTT	TCTTCTCTGGGTTGGCACACACTT
CXCL1	TGCACCCAAACCGAAGAAGTC	CAAGGGAGCTTCAGGGTGAAG
CXCL2	GCGCTGTCAATGCCTGAAGTC	CGTCACACTCAAGCTCTGGAT
CXCL10	CCAAGTGCTGCCGTCATTTTC	GGCTCGCAGGGATGATTTCAA
5-LO	ACTACATCTACCTCAGCCTCATT	GGTGACATCGTAGGAGTCCAC
12/15-LO	GGCTCCAACAACGAGGTCTAC	AGGTATTCTGACACATCCACCTT
COX-2	TTCAACACACTCTATCACTGGC	AGAAGCGTTTGCGGTACTCAT
Cmklr1	ATGGAGTACGACGCTTACAACG	GGTGGCGATGACAATCACCA
Fpr2	GAGCCTGGCTAGGAAGGTG	TGCTGAAACCAATAAGGAACCTG
Lgr6	CTCACCGAGATCCCCGTCA	TGCGGTTGTTATGTAGATGCAG
Gpr18	CACCCTGAGCAATCACAACCA	AGTGACATTAACAAACAGCCCA
MMP2	CAAGTTCCCCGGCGATGTC	TTCTGGTCAAGGTCACCTGTC
MMP3	ACATGGAGACTTTGTCCCTTTTG	TTGGCTGAGTGGTAGAGTCCC
MMP7	CTGCCACTGTCCCAGGAAG	GGGAGAGTTTTCCAGTCATGG
MMP8	TCTTCCTCCACACACAGCTTG	CTGCAACCATCGTGGCATTC
MMP9	GGACCCGAAGCGGACATTG	CGTCGTCGAAATGGGCATCT
MMP10	GAGCCACTAGCCATCCTGG	CTGAGCAAGATCCATGCTTGG
MMP11	CCGGAGAGTCACCGTCATC	GCAGGACTAGGGACCCAATG
MMP12	AATGCTGCAGCCCCAAGGAAT	CTGGGCAACTGGACAACTCAA
MMP13	CTTCTTCTTGTTGAGCTGGACTC	CTGTGGAGGTCACTGTAGACT
TIMP1	CCTAGAGACACACCAGAGCA	TACCGGATATCTGCGGCATT
TIMP2	GGCAACCCCATCAAGAGGA	CCTTCTGCCTTTCCTGCAATTAG
TIMP3	CTTCTGCAACTCCGACATCGT	GGGGCATCTTACTGAAGCCTC
TIMP4	TGTGGCTGCCAAATCACCA	TCATGCAGACATAGTGCTGGG
F4/80	CCCCAGTGTCCTTACAGAGTG	GTGCCCAGAGTGGATGTCT
CD68	CTTCCCACAGGCAGCACAG	AATGATGAGAGGCAGCAAGAG G
CD64	GTCGGTGGGGAAGTGGTTAAT	CCCCTCACACCATAAAGTGAC
Mpo	AGTTGTGCTGAGCTGTATGGA	CGGCTGCTTGAAGTAAAACAGG
Elane	TTGCCAGGAATTTCGTCATGT	GTTGGCGTTAATGGTAGCGGA
S100a8	GTAGAGGGCATGGTGATTTCCT	CCGTCTTCAAGACATCGTTTGA
Hmox1	AAGCCGAGAATGCTGAGTTCA	GCCGTGTAGATATGGTACAAGGA
Nqo1	AGGATGGGAGGTACTCGAATC	AGGCGTCCTTCCTTATATGCTA
Prdx1	AATGCAAAAATTGGGTATCCTGC	CGTGGGACACACAAAAGTAAAGT
L32	TTAAGCGAAACTGGCGGAAAC	TTGTTGCTCCCATAACCGATG

Supplemental Table 5. Sequences of primers used in this study. The primers were synthesized by Integrated DNA Technologies. L32, 60S ribosomal protein L32, was used as internal control.
